# From biological evidence to clinical application: a scoping review of oral fluid biomarkers in type 2 diabetic periodontitis

**DOI:** 10.3389/froh.2026.1892626

**Published:** 2026-07-28

**Authors:** Julie Toby Thomas, Betsy Joseph, Nur R. A. S. Aji, Toby Thomas, Timo Sorsa, Sukumaran Anil, Tuomas Waltimo

**Affiliations:** 1 Department of Oral and Maxillofacial Diseases, Helsinki University and University Hospital, Helsinki, Finland; 2 Department of Periodontics, Saveetha Dental College and Hospitals, Saveetha Institute of Medical and Technical Sciences, Chennai, India; 3 Department of Periodontics, Faculty of Dentistry, Universitas Gadjah Mada, Yogyakarta, Indonesia; 4 Department of Restorative Dentistry, College of Dentistry, Majmaah University, Al-Majmaah, Saudi Arabia; 5 Division of Periodontology, Department of Dental Medicine, Karolinska Institutet, Stockholm, Sweden; 6 Center of Excellence in Precision Medicine and Digital Health, Department of Physiology, Faculty of Dentistry, Chulalongkorn University, Bangkok, Thailand; 7 Global Research Cell, Dr. D. Y. Patil Dental College and Hospital, Dr. D. Y. Patil Vidyapeeth, Pune, India; 8 Department of Medicine, University of Basel, Basel, Switzerland

**Keywords:** cytokines, gingival crevicular fluid, matrix metalloproteinase-8 (MMP-8), oral-fluid biomarkers, periodontitis, point-of-care testing, saliva, type 2 diabetes mellitus (T2DM)

## Abstract

**Background:**

Diabetes mellitus and periodontitis share a bidirectional inflammatory relationship. Oral fluids provide non-invasive access to host and microbial signals relevant to both diseases, but clinical translation of oral fluid biomarkers in diabetic periodontitis remains limited.

**Objectives:**

To map the evidence on salivary, gingival crevicular fluid (GCF), and oral rinse biomarkers in adults with type 2 diabetes mellitus and periodontitis, emphasizing analytical platforms, diagnostic performance, and chair-side readiness.

**Materials and methods:**

PubMed/MEDLINE, Scopus, Web of Science, Embase, and the Cochrane Library were searched from 2005 to April 2026. Forward and backward citation tracking supplemented the search. Peer-reviewed human studies involving adults with physician-diagnosed diabetes and clinically assessed periodontitis reporting quantitative oral fluid biomarkers were included. Eligible studies examined associations with glycemic status, periodontal parameters, or diagnostic accuracy. Intervention studies were excluded. Two reviewers independently screened records. A pre-specified charting form captured study design, oral fluid type, biomarker class, assay platform, and diagnostic performance. Findings were synthesized narratively.

**Results:**

Diabetic periodontitis was associated with elevated active-matrix metalloproteinase-8 (aMMP-8), MMP-9, interleukin-1 beta (IL-1β), IL-6, advanced glycation end products, oxidative stress markers, selected microRNAs, and disrupted MMP/TIMP (tissue inhibitor of metalloproteinases) balance. Among single analytes, aMMP-8 and IL-1β were the most consistently reported. aMMP-8 discriminated periodontitis [area under the curve (AUC) 0.84 in oral rinse and 0.81 in saliva], whereas IL-1β showed high reported accuracy (AUC 0.87–0.93). Multi-marker panels generally achieved higher accuracy (AUC up to 0.92–1.00). Among point-of-care approaches, aMMP-8 had the most extensive chair-side validation. Due to substantial heterogeneity in study design, reference standards, and clinical objectives, findings are presented by intended application (diagnosis, differentiation of diabetic from non-diabetic periodontitis, severity assessment, glycemic assessment, monitoring, and screening) rather than direct biomarker comparison.

**Conclusions:**

Oral fluid biomarkers, particularly aMMP-8, show reproducible associations with glycemic progression and periodontal tissue destruction in type 2 diabetic periodontitis and demonstrate promise for non-invasive screening and monitoring. Standardization of sampling protocols, assay thresholds, and biomarker panels is required before routine clinical implementation.

## 1 Introduction

Diabetes mellitus and periodontitis share a bidirectional inflammatory relationship that now shapes guideline development in both dental and primary care. Pooled epidemiological data indicate periodontitis affects roughly twice as many adults with diabetes as their non-diabetic peers (1). The reverse direction is also supported, with higher diabetes prevalence documented among adults attending periodontal care (2). Recent European Federation of Periodontology and WONCA Europe consensus statements frame periodontitis alongside cardiovascular disease as a comorbidity of diabetes (3). Non-surgical periodontal therapy yields small but consistent improvements in glycated hemoglobin in patients with type 2 diabetes (T2DM) (4).

**Figure 1:**
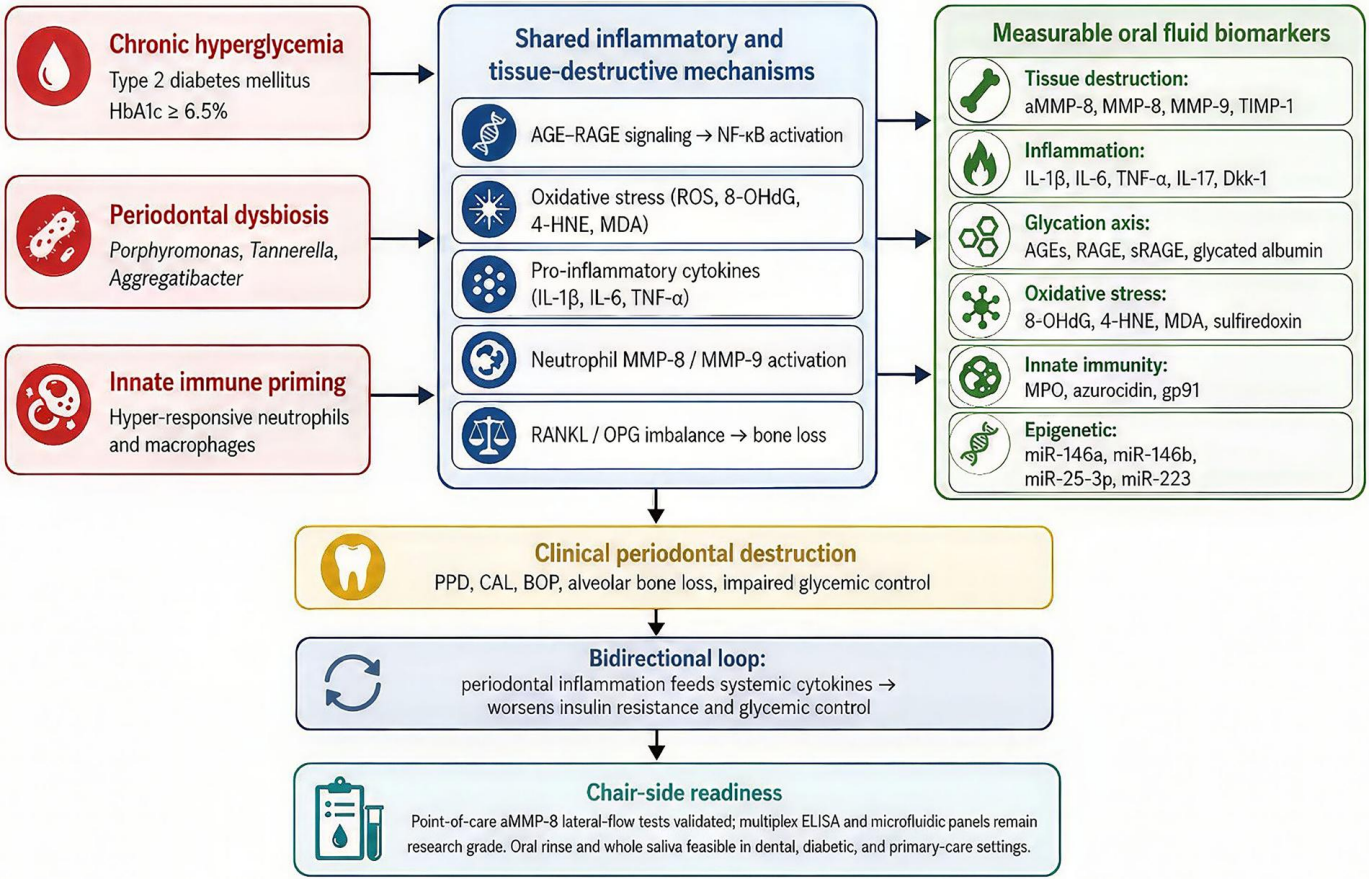
Biological mechanisms linking type 2 diabetes mellitus and periodontitis, and their associated oral fluid biomarkers. Type 2 diabetes mellitus (T2DM) and periodontitis exhibit a bidirectional relationship. T2DM exacerbates periodontitis through hyperglycemia-induced advanced glycation end-products (AGEs)/RAGE signaling, oxidative stress, impaired neutrophil function, and heightened pro-inflammatory cytokine responses (e.g., IL-1β, IL-6, TNF-α), leading to enhanced tissue destruction and alveolar bone loss. Conversely, periodontitis can worsen glycemic control via systemic inflammation and dissemination of periodontal pathogens or their virulence factors. Associated oral fluid biomarkers include elevated levels of MMP-8, MMP-9, IL-1β, IL-6, resistin, and 8-OHdG, reflecting intensified inflammation, tissue breakdown, and oxidative stress in patients with both conditions.

Chronic hyperglycemia and periodontal inflammation are associated with dysregulated immune responses, oxidative stress, tissue destruction, and altered host-microbial interactions, resulting in measurable changes in oral-fluid biomarkers (5–11). These biomarkers, detectable in saliva, gingival crevicular fluid (GCF), oral rinse, and peri-implant sulcular fluid, include matrix metalloproteinases (MMPs), cytokines, oxidative stress mediators, glycation-related molecules, and microRNAs (12–14). Their expression has been associated with both periodontal severity and glycemic status, supporting their potential utility in disease monitoring and risk assessment. The major biological pathways linking hyperglycemia, periodontal inflammation, and oral-fluid biomarkers are summarized in Figure 1.

Oral fluid compartments such as saliva, GCF, and oral rinse are increasingly used for biomarker analysis in periodontal and diabetes research. Whole unstimulated saliva reflects whole-mouth inflammatory burden and is the most practical matrix for both dental and medical settings. GCF provides site-specific host and microbial information but requires technique-sensitive sampling, while oral rinse offers a pooled and standardized sample suitable for chair-side screening and primary care workflows. Point-of-care (POC) active matrix metalloproteinase (aMMP-8) testing has also demonstrated clinical utility in this population and supports chair-side decision-making (15).

Despite a growing biomarker literature, clinical translation is limited by heterogeneous analytes, inconsistent cut-offs, and few head-to-head platform comparisons. Periodontitis remains underdiagnosed in diabetes care pathways, and integrated oral-systemic screening is uncommon (16, 17). Guideline integration across dental, diabetes, and primary care is uneven and has not been mapped comprehensively (18). Although earlier reviews have examined the biological mechanisms linking periodontitis and type 2 diabetes, the rapid emergence of multi-omics profiling, the expansion of chair-side point-of-care platforms, and the 2024 European Federation of Periodontology and WONCA Europe consensus, which frames periodontitis alongside cardiovascular disease as a comorbidity of diabetes (3), together warrant an updated map that connects this biological evidence to clinical application. Because the diabetes and periodontitis relationship is increasingly understood within a broader cardiometabolic risk profile, this review also considers the cardiovascular and cardiometabolic relevance of the biomarkers mapped. Therefore, this scoping review aims to map the biological-to-clinical translation landscape of oral-fluid biomarkers in diabetic periodontitis. The guiding Population-Concept-Context (PCC) question was: among adults with T2DM and periodontitis, which oral-fluid biomarkers and assay platforms have been evaluated for their association with glycemic status, periodontal status, and/or diagnostic accuracy across dental, medical, and community care settings?

## 2 Methods

### 2.1 Protocol and registration

This scoping review followed the Joanna Briggs Institute methodology for scoping reviews (19) and is reported in accordance with the PRISMA-ScR checklist (20). A completed PRISMA-ScR checklist is provided as Supplementary Material S1*.* The review framework, comprising the Population, Concept, and Context eligibility criteria and the biological-to-clinical synthesis axis, was defined *a priori* before the search was executed; no changes were made to the eligibility criteria or synthesis categories after screening began.

### 2.2 Eligibility criteria

The review used the Population, Concept, Context (PCC) framework. *Population:* adults aged 18 years and older with a physician-confirmed diagnosis of T2DM and a clinical diagnosis of periodontitis by the attending dental or research team. *Concept:* any quantitatively reported oral fluid biomarker, including salivary, GCF, oral rinse, or peri-implant sulcular fluid (PISF) analytes spanning host enzymes, inflammatory cytokines, oxidative stress mediators, advanced glycation end products and their receptor, bone metabolism regulators, innate immune markers, microRNAs, and multi-marker panels. *Context:* primary care, dental, hospital, or community care settings reporting at least one of the following: association with periodontal clinical parameters like probing pocket depth (PPD), clinical attachment loss (CAL), bleeding on probing (BoP), association with glycemic control (HbA1c, fasting plasma glucose), diagnostic performance metrics. Studies spanning the dysglycemia continuum, including participants with prediabetes, were eligible where they reported data in adults with type 2 diabetes, and studies enrolling mixed type 2 diabetes and prediabetes populations were included when type 2 diabetes participants constituted the majority or type 2 diabetes subgroup analyses were reported; findings specific to prediabetes are identified as such in the synthesis. Across the included studies, periodontitis was defined using either the 2018 AAP/EFP staging and grading classification or earlier diagnostic criteria (e.g., the 2012 CDC/AAP case definition, the 2007 Page and Eke case definitions, or the 1999 Armitage classification) (21–23). The periodontitis case definition used in each included study is provided in Supplementary Material S3.

*Study designs:* Primary human studies, including cross-sectional, case-control, prospective or retrospective cohort, and POC validation studies. Conference abstracts were eligible only when extractable data were verifiable through a linked full report. *Exclusions:* animal or *in vitro* studies, narrative, scoping, or systematic reviews (retained only for cross-reference), pediatric populations, studies of T1DM alone, and studies that did not report at least one oral fluid measurement.

### 2.3 Information sources

Five electronic bibliographic databases were searched: PubMed/MEDLINE (National Library of Medicine), Scopus (Elsevier), Web of Science Core Collection (Clarivate), Embase (Elsevier), and the Cochrane Library (Wiley). Grey literature was retrieved from Google Scholar (first 200 ranked results), ClinicalTrials.gov, and the World Health Organization International Clinical Trials Registry Platform. Forward and backward citation tracking was applied to all full text included studies. Table-of-contents hand searches of the Journal of Periodontology, Journal of Clinical Periodontology, Journal of Periodontal Research, and Oral Diseases from 2020 to April 2026 supplemented the database output.

### 2.4 Search strategy

Searches were run from January 1, 2005, through April 15, 2026. No language filter was applied at the database level; non-English records were translated when abstracts indicated potential eligibility. The complete PubMed/MEDLINE search string is shown below:

(“diabetes mellitus”[MeSH] OR “diabetes mellitus, type 2”[MeSH] OR “type 2 diabetes”[tiab] OR “T2DM”[tiab] OR diabet*[tiab]) AND (“periodontitis”[MeSH] OR “periodontal diseases”[MeSH] OR periodontitis[tiab] OR “periodontal disease*”[tiab]) AND (“saliva”[MeSH] OR “gingival crevicular fluid”[MeSH] OR saliv*[tiab] OR “GCF”[tiab] OR “oral fluid*”[tiab] OR “oral rinse”[tiab] OR mouthwash[tiab]) AND (“biomarkers”[MeSH] OR biomark*[tiab] OR “matrix metalloproteinase*”[tiab] OR “MMP-8”[tiab] OR “aMMP-8”[tiab] OR cytokin*[tiab] OR interleukin*[tiab] OR “oxidative stress”[tiab] OR “advanced glycation end*”[tiab] OR microRNA*[tiab] OR miR-*[tiab]).

Adapted search strings for Scopus, Web of Science, Embase, and the Cochrane Library are provided in Supplementary Material S2*.* The combined search returned 178 records before deduplication, of which 14 were duplicates; the per-database record counts are reported in Supplementary Material S2*.* The focused yield reflects the deliberately specific intersection of diabetes, periodontitis, oral fluid, and biomarker terms; the sensitivity of this strategy is considered in the Limitations.

### 2.5 Selection of sources of evidence

**Figure 2:**
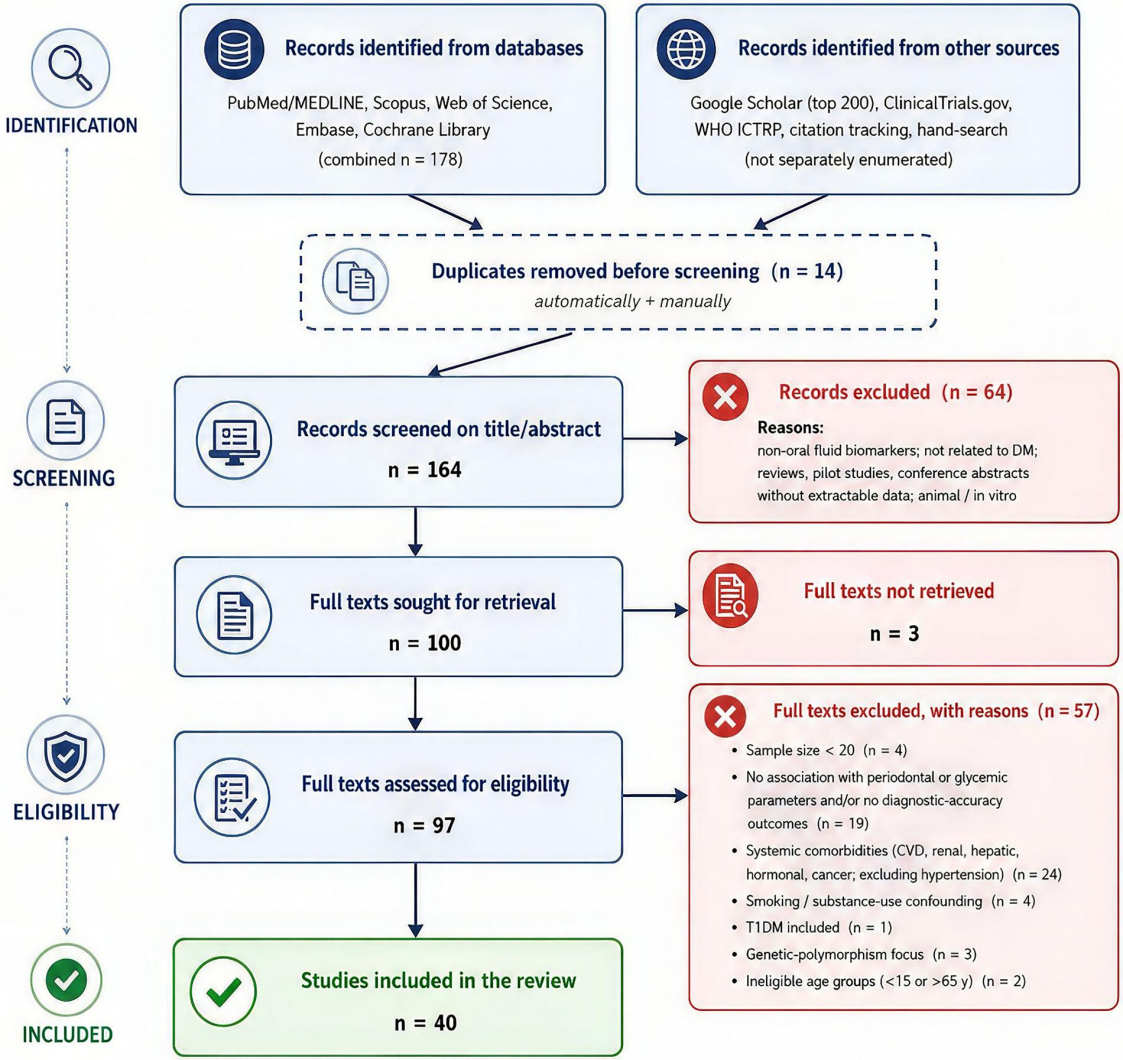
PRISMA flow diagram illustrating the study selection process. Adapted from Page et al. (2021) for PRISMA-ScR reporting (Tricco et al., 2018).

Records were exported to the reference manager, and duplicates were removed before screening. Two independent reviewers screened titles and abstracts against the eligibility criteria. Full texts of potentially eligible records were retrieved and independently screened by the same reviewers. Disagreements at either stage were resolved through discussion; unresolved cases were adjudicated by a third reviewer. A PRISMA-ScR flow diagram reports record counts at each stage (Figure 2). Agreement between the two reviewers was high at both the title and abstract and the full-text screening stages.

### 2.6 Data charting process

A pre-specified charting form was developed in Microsoft Excel and pilot-tested on ten included studies. The form was refined iteratively in line with JBI guidance (19). Two reviewers independently extracted data from each included study, and discrepancies were reconciled by consensus. The charting form is provided as a Supplementary Material to support reproducibility.

### 2.7 Data items

Charted items included: first author, publication year, country, study design, sample size, participant demographics, diabetes diagnosis and glycemic status, periodontal case definition, oral fluid matrix (saliva, GCF, oral rinse, PISF), biomarker(s) measured, assay platform (enzyme-linked immunosorbent assay, multiplex immunoassay, POC test, quantitative polymerase chain reaction, mass spectrometry), reported cut-off values, reported diagnostic performance [sensitivity, specificity, area under the receiver operating characteristic (ROC) curve], association with periodontal clinical parameters, association with glycemic control, and clinical translation or implementation outcomes.

### 2.8 Critical appraisal of sources of evidence

Consistent with JBI scoping review methodology, a formal risk-of-bias appraisal was not performed (19). Major methodological limitations of each study, including sample size, adjustment for confounders, and assay validation, were noted narratively when they affected interpretation.

### 2.9 Synthesis of results

The charting form, the *a priori* PCC review question, and the synthesis were linked through an explicit analytical framework. Charted items were first classified by the clinical objective each study addressed, defined by its reference standard: (i) diagnosis of periodontitis (periodontal health vs. periodontitis), (ii) differentiation of diabetic from non-diabetic periodontitis (diabetes or metabolic status within periodontitis), (iii) assessment of periodontal disease severity (probing pocket depth, clinical attachment loss, bleeding on probing, and staging), (iv) glycemic assessment (HbA1c or glycemic-control category), (v) treatment response and disease monitoring (within-patient baseline vs. post-therapy), and (vi) screening or case-finding in integrated dental-medical care (detection of previously undiagnosed dysglycemia or periodontitis). Within each objective, biomarker findings were synthesized narratively and interpreted only against the corresponding reference standard; diagnostic-accuracy estimates, correlations with periodontal parameters, and associations with glycemic markers were treated as distinct outcomes and were not pooled or compared across objectives. The biological pathways underlying these associations were mapped separately along a biological-to-clinical axis and are presented in the Discussion. The resulting chain, from the predefined review question through data charting and objective-based synthesis to the translational-readiness appraisal, is reported so that the derivation of the framework is transparent. A clinical translation readiness appraisal (Table 1) and an objective-to-reference-standard map (Table 2) are presented alongside the biological evidence (Tables 3, 4).

**Table 1:** Translational readiness of the principal oral fluid biomarker classes for diabetic periodontitis, appraised against six biomarker-development criteria.

Biomarker/class	Analytical validity	Diagnostic validity	External validation	Chair-side feasibility	Cost-effectiveness	Regulatory/pathway	Primary clinical objective (reference standard)
aMMP-8 (point-of-care)	Established (IFMA/ELISA/POC; cut-off 20 ng/mL)	Established (AUC 0.75 to 0.89)	Emerging (multiple cohorts)	Established (commercial chair-side test)	Not yet established	Emerging	Screening and monitoring; diagnosis (periodontitis vs. health)
IL-1β	Established (ELISA; multiplex)	Established (AUC 0.87 to 0.98)	Limited	Emerging (multiplex chair-side, e.g., ORALyzer)	Not yet established	Not yet	Diagnosis (periodontitis vs. health); severity
IL-6	Established (ELISA)	Moderate (AUC 0.79 to 0.86)	Limited	Emerging	Not yet established	Not yet	Glycemic assessment (HbA1c); severity adjunct
AGE/RAGE/sRAGE	Emerging (ELISA)	Moderate; independent link to HbA1c	Limited	Not yet	Not yet established	Not yet	Glycemic assessment (HbA1c)
Oxidative stress (8-OHdG, 4-HNE, MDA)	Emerging (ELISA, spectrophotometry)	Moderate alone; high in panels (AUC up to 0.92 to 1.00)	Limited	Not yet	Not yet established	Not yet	Differentiation (diabetic vs. non-diabetic); severity
Metabolomic/microbiome	Limited (LC-MS, 16S; infrastructure-heavy)	High in early studies (AUC up to 0.93)	Not yet	Not yet	Low feasibility	Not yet	Glycemic assessment (glycemic-control state)
microRNA	Limited (RT-qPCR)	High in pilot studies (AUC >0.9 for some)	Not yet	Not yet	Not yet established	Not yet	Diagnosis and differentiation (diabetic vs. non-diabetic)

AUC, area under the receiver operating characteristic curve; aMMP-8, active matrix metalloproteinase-8; AGE, advanced glycation end products; ELISA, enzyme-linked immunosorbent assay; HbA1c, glycated hemoglobin; IFMA, immunofluorometric assay; IL, interleukin; LC-MS, liquid chromatography-mass spectrometry; MDA, malondialdehyde; RAGE, receptor for advanced glycation end products; RT-qPCR, reverse transcription quantitative polymerase chain reaction; sRAGE, soluble RAGE; 4-HNE, 4-hydroxy-2-nonenal; 8-OHdG, 8-hydroxy-2′-deoxyguanosine.

**Table 2:** Mapping of oral fluid biomarkers to clinical objectives and their reference standards.

Clinical objective	Reference standard	Representative biomarkers/panels	Illustrative performance from included studies
Diagnosis of periodontitis	Periodontal health vs. periodontitis	aMMP-8; IL-1β; IL-6; asprosin; MMP-8/TIMP-1 and multimarker panels; salivary miRNAs	aMMP-8 AUC 0.75–0.84; IL-1β AUC 0.87–0.93; multimarker panels AUC up to 0.95
Differentiation of diabetic vs. non-diabetic periodontitis	Diabetes or metabolic status within periodontitis	aMMP-8; IL-1β; oxidative-glycation panels; AGE; 4-HNE; miRNAs	IL-1β AUC 0.872–0.975; oxidative/glycation panel AUC 0.92–1.00; aMMP-8 metabolic vs. non-metabolic AUC 0.43–0.68
Assessment of periodontal severity	PPD, CAL, BoP, and staging	aMMP-8/MMP-8; IL-1β; 8-OHdG and 8-IsoP; AGE; miR-223/miR-200b	Correlations r ≈ 0.20–0.99 with periodontal parameters
Glycemic assessment	HbA1c or glycemic-control category	Salivary RAGE; glycated albumin; IL-6; microbiome-metabolome panels	RAGE independent HbA1c predictor (R² = 0.509); salivary metabolite panel AUC 0.9271; bacterial panel AUC 0.9151
Treatment response and monitoring	Within-patient baseline vs. post-therapy	aMMP-8 (from broader literature; intervention studies excluded from scope)	Not charted within scope; identified as an evidence gap
Screening and case-finding	Detection of undiagnosed dysglycemia or periodontitis	aMMP-8 point-of-care plus clinical risk factors	AUC 0.683 → 0.759; combined point-of-care model AUC 0.713

Performance values are illustrative estimates drawn from individual included studies and are reported within each objective; they are not pooled and are not comparable across objectives.

**Table 3:** Human evidence for oral fluid biomarkers associated with periodontitis in type 2 diabetes Mellitus.

Biomarker group	Biomarker(s)	Oral fluid/sample	Assay method	Key findings (references)
Tissue-destructive enzymes	aMMP-8/MMP-8	Saliva, oral rinse, GCF	IFMA, ELISA, POC	Markedly elevated with a disease gradient (DP > non-DP > healthy, *p* < 0.001); correlates with BOP, PPD, CAL (*r* = 0.29–0.77); reflects active collagen breakdown; enhanced by hyperglycemia. aMMP-8 strongly associated with prediabetes (*ρ* = 0.646) and decreases after periodontal therapy independent of HbA1c (14, 15, 30, 31, 33, 39, 54, 69)
	MMP-9	Saliva, GCF	ELISA, zymography Multiplex/Luminex	Significantly elevated (*p* < 0.001); correlates strongly with aMMP-8 (*ρ* = 0.721); reflects coordinated collagenolytic and gelatinolytic activity; primarily linked to local inflammation (32, 33, 42, 69). No significant correlations between MMP-9 or MPO and periodontal or glycemic parameters in diabetic groups (61).
	TIMP-1	Saliva, GCF	ELISA	Regulatory inhibitor insufficient to counteract elevated MMPs; MMP-8/TIMP-1 ratio markedly increased; strong correlation with aMMP-8 (*ρ* = 0.879), indicating protease-inhibitor imbalance (12, 33).
	MMP-2/TIMP-1	Whole saliva	Gelatin zymography, Western blot	MMP-2 activity increased with periodontal severity (*p* < 0.05). TIMP-1 elevated in periodontitis but reduced under poor glycemic control. Poor glycemia associated with reduced MMP-2 and TIMP-1, indicating dysregulated matrix turnover rather than simple upregulation (40).
Inflammatory cytokines	IL-1β	Saliva, GCF	ELISA	Strongly elevated (3.82 vs. 2.35; *p* = 0.0005); correlates with PD and BOP (*r* = 0.49–0.71); key mediator of diabetes-associated periodontal inflammation (12, 14, 25, 39, 47). Periodontitis showed stronger effects on βG (β = 0.75) and IL-1β (β = 2.037) than diabetes (βG β = 0.407), while diabetes had no significant effect on IL-1β (*p* = 0.497) (41).
	IL-6	Saliva, GCF	ELISA	Moderately elevated; correlates with HbA1c (*r* = 0.60) and periodontal severity (*r* = 0.18–0.32); weaker association with clinical indices; reflects metabolic-inflammatory link (14, 26, 42).
	TNF-α, IL-10, IL-6, IL-8, MIP-1α, MIP-1β, RANTES, IL-7, G-CSF, VEGF	GCF	Multiplex immunoassay/ELISA-based	DM + CP showed ↑ IL-8 and MIP-1β and ↓ TNF-α, IL-4, IFN-*γ*, RANTES, IL-7 vs. CP. Compared to DM, DM + CP had ↓ IL-6, IL-7, G-CSF. DM group showed ↑ IL-10, VEGF, G-CSF vs. CP. Both diabetes groups showed ↓ MIP-1α and FGF vs. CP. HbA1c showed weak positive correlation with pro-inflammatory cytokines (*r* = 0.27, *P* = 0.02) (52).
	IL-17/Del-1 axis	GCF	ELISA	Salivary IL-17 was elevated (highest in T2DM+CP) and Del-1 reduced in disease groups (*P* < 0.05). IL-17 showed a positive association with disease severity (*r* = 0.329–0.645), while Del-1 demonstrated consistent inverse correlations (r = -0.57 to −0.66) across periodontal parameters and glycemic control, indicating a pro-inflammatory imbalance. Reduced Del-1 (OR = 0.369) and increased IL-17 (OR = 1.654) were independent risk factors for periodontitis in T2DM (43, 44).
	MIP-1α, IFN-α, BAFF	Saliva, GCF	ELISA	Elevated; stronger associations with microbial dysbiosis and disease severity in diabetes (12, 47).
Glycemic & metabolic markers	AGE/RAGE/sRAGE	Saliva, GCF, PISF	ELISA	AGE and RAGE increased; sRAGE decreased; correlate with HbA1c and periodontal parameters (*r* = 0.35–0.51); RAGE independently predicts HbA1c (β ≈ 0.161; R² = 0.509) (24, 34, 63). AGEs in PISF were significantly higher in prediabetes and T2DM compared with controls (*P* < .05 and *P* < .01) and higher in T2DM than prediabetes (*P* < .05), with positive correlations with PD (*P* = 0.0371) and marginal bone loss (MBL) (*P* = 0.0117), and a negative correlation with plaque index (PI) (*P* = 0.0276) (51).
	C-peptide, insulin, GIP, GLP-1, glucagon, ghrelin, leptin, PAI-1, visfatin	GCF	Multiplex biometric immunoassay	PD ≥4 mm correlated with C-peptide (r = −0.240, *P* < 0.01), GIP (r = 0.255, *P* < 0.01), and visfatin (r = 0.241, *P* < 0.01). Periodontitis associated with altered glucoregulatory biomarker profile in T2DM and non-diabetic subjects (52).
Oxidative stress & antioxidant balance	MDA, 4-HNE, 8-OHdG	Saliva, GCF	ELISA, spectrophotometry	Elevated with highest levels in diabetic periodontitis; correlate with BOP and CAL (r = 0.48–0.49); stronger associations in GCF under hyperglycemia (29, 35, 48, 49).
	SOD, CAT, GR	Saliva	Enzymatic assays	Reduced activity; inversely correlated with periodontal destruction; more pronounced in diabetes (48, 49).
	NO, PMN cells	Saliva, blood	Griess assay, microscopy	Nitric oxide levels significantly increased in poor glycemic control (*p* < 0.05). PMN counts elevated with worsening glycemic status, reflecting enhanced systemic and local inflammatory response (40).
	8-IsoP	Saliva	Immunoassay (8-IsoP assay)	CP with DM showed lowest oxidative stress levels among diseased groups. 8-IsoP showed strong positive correlation with GI, PPD, and CAL (*P* < 0.001) (55).
	TAOC, TOS, OSI	Saliva	spectrophotometric assays	PH showed highest TAOC, while GCP, DM, and especially GCP-DM showed elevated TOS and OSI, indicating increased oxidative stress (49).
	TOS, sulfiredoxin	GCF	ELISA (sulfiredoxin), colorimetric assay (TOS)	TOS increased in periodontitis and highest in PSII with DM (*P* < 0.001). A strong positive correlation observed between TOS and sulfiredoxin in diabetic periodontitis (*r* = 0.65, *P* < 0.005), with HbA1c and TOS identified as significant predictors (36).
	MPO	Saliva GCF	ELISA, Multiplex/Luminex	Elevated; correlates with periodontal inflammation and oxidative stress (65). No significant correlations between MPO and periodontal or glycemic parameters in diabetic groups (61).
Innate immunity markers	Calprotectin	GCF	ELISA	Reflects neutrophil activation; limited association with HbA1c (56).
	AIM2, IFI16; IL-18	Unstimulated whole saliva	ELISA	AIM2, IFI16, and IL-18 levels were highest in T2DM with periodontitis. Strong positive correlations were observed with glycemic and periodontal parameters: HbA1c (AIM2: *r* = 0.788; IL-18: *r* = 0.782), FBG (AIM2: *r* = 0.664; IL-18: *r* = 0.685), BOP (IL-18: *r* = 0.768). AIM2 showed strong correlation with IL-18 (*r* = 0.894) (45).
	β-glucuronidase	Unstimulated saliva	Fluorometric assay (βG), ELISA (IL-1β)	Diabetes independently increased βG but not IL-1β, indicating periodontal disease as the dominant driver of oral inflammatory burden (41).
Epigenetic markers	miR-223/miR-200b	GCF	RT-PCR	Upregulated; correlate with CAL, PD, TNF-α; stronger associations in T2DM; miR-223 shows highest correlation (*r* = 0.62–0.67) (37, 38).
Microbiome & metabolome signatures	Oral microbiome shifts	Saliva, GCF	16S rRNA sequencing	Strong positive correlations between salivary and GCF abundance of key periodontal species (r² = 0.095–0.54; *p* < 0.001), supporting saliva as a reliable surrogate for subgingival microbiota. Dysbiosis increases with poor glycemic control, with enrichment of periodontopathogens (*P. gingivalis*, *Prevotella spp.*, *T. denticola*) and reduced health-associated taxa (*Neisseria elongata*). High diagnostic accuracy for glycemic status (AUC = 0.915) (12).
	Salivary bacterial species, metabolites, diversity indices.	Saliva, GCF	16S rRNA sequencing, LC-MS metabolomics	Significant positive correlations were observed between salivary and GCF microbial species, along with strong associations between differential bacteria and metabolites, indicating coordinated microbial-metabolic interactions linked to glycemic status in T2DM-associated periodontal disease (58).

AGEs, Advanced glycation end products; AIM2, Absent in melanoma 2; aMMP-8, Active matrix metalloproteinase-8; BAFF, B-cell activating factor; BOP, bleeding on probing; CAL, clinical attachment level; CAT, catalase; C-peptide, connecting peptide (proinsulin marker); CP, chronic periodontitis; DM, diabetes mellitus; T2DM, type 2 diabetes mellitus; ELISA, enzyme-linked immunosorbent assay; FBG, fasting blood glucose; GCF, gingival crevicular fluid; GIP, glucose-dependent insulinotropic polypeptide; GLP-1, glucagon-like peptide-1; GR, glutathione reductase; HbA1c, glycated hemoglobin; IFMA, immunofluorometric assay; IFI16, interferon gamma inducible protein 16; IFN-α/*γ*, interferon alpha/gamma; IL, interleukin; LC-MS, liquid chromatography-mass spectrometry; MDA, malondialdehyde; miR/miRNA, microRNA; MIP-1α/β, macrophage inflammatory protein-1 alpha/beta; MMP, matrix metalloproteinase; MPO, myeloperoxidase; NF-*κ*B, nuclear factor kappa B; NO, nitric oxide; PAI-1, plasminogen activator inhibitor-1; PD, periodontal disease; PPD, probing pocket depth; PISF, peri-implant sulcular fluid; PMN, polymorphonuclear neutrophils; RAGE, receptor for advanced glycation end products; RANKL, receptor activator of nuclear factor kappa-B ligand; RANTES, regulated upon activation, normal T-cell expressed and secreted; ROS, reactive oxygen species; RT-PCR, reverse transcription polymerase chain reaction; sRAGE, soluble receptor for advanced glycation end products; SOD, superoxide dismutase; TAOC, total antioxidant capacity; TIMP-1, tissue inhibitor of metalloproteinases-1; TOS, total oxidative stress; TNF-α, tumor necrosis factor alpha; VEGF, vascular endothelial growth factor; βG, beta-glucuronidase.

**Table 4:** Clinical translation and implementation readiness of oral fluid biomarkers for diabetic periodontitis.

Category	Biomarker(s)	Study (Author, Year)	Study design	Diabetic population (*n*, type)	Oral fluid	Assay platform	Clinical application	Diagnostic performance (Sens/Spec/AUC/Cut-offs)	Clinical utility	Implementation considerations
Tissue-destructive enzymes	aMMP-8	Grigoriadis A et al., 2021	Cross-sectional clinical study	69; Periodontal clinic patients with normoglycemia and prediabetes (HbA1c 5.7%–6.4%)	Oral rinse	Chair-side aMMP-8 immunotest + chair-side HbA1c PoCT	Identification of undiagnosed dysglycaemia risk in periodontal patients	AUC: 0.759 (BMI + age ≥45 + aMMP-8 model); baseline model AUC: 0.683; significant association with prediabetes (*p* < 0.05); periodontal disease association also significant (*p* < 0.05) (chair-side aMMP-8 cut-off 20 ng/mL)	Demonstrates incremental diagnostic value of aMMP-8 beyond conventional risk factors	Non-invasive chair-side tool; enhances predictive models; supports opportunistic screening in dental clinics; requires integration with metabolic risk assessment protocols
		Thomas JT et al., 2025	Cross-sectional diagnostic accuracy study	40; Metabolic syndrome with PD	Oral rinse, saliva, GCF	ELISA	Assessment of periodontitis in metabolic syndrome and evaluation of systemic inflammatory burden	Oral rinse: AUC 0.89; saliva: AUC 0.85; GCF: AUC 0.82; cut-off 20 ng/mL	High diagnostic accuracy for early detection and monitoring of periodontitis in MetS patients	Oral rinse showed the highest diagnostic performance, followed by saliva and GCF; 20 ng/mL cut-off provides optimal sensitivity and specificity; standardization required for clinical implementation
Inflammatory cytokines	IL-6	Sangappa M et al., 2024	Cross-sectional	120; T2DM -PD with and without tooth loss	Unstimulated saliva	ELISA	Detection and assessment of CP severity	Sens 53.3%; Spec 68.6%; Accuracy 60%	Adjunct inflammatory marker	Moderate performance; lacks disease specificity
	Asprosin, IL-1β, IL-39, IL-40	Oruc AH & Babayiğit O, 2026	Cross-sectional	88; T2DM and non-T2DM	Unstimulated saliva	ELISA	Non-invasive periodontal inflammation detection	IL-1β AUC 0.975 (cut-off 1228.5 pg/mL); IL-40 AUC 0.750; Asprosin AUC 0.804	Identifies periodontal-metabolic inflammation	ELISA-dependent; IL-39 non-informative
Oxidative stress & inflammatory panel	8-OHdG, MDA, 4-HNE, AGE, RAGE, hsCRP	Altıngöz et al., 2021	Cross-sectional multi-group study	121; T2DM-PH, T2DM-PD, SH-PH, SH-PD	Saliva	ELISA	Periodontitis screening in diabetes	Best single markers: 4-HNE & RAGE in DM: AUC 0.85, Sens 0.80–0.83, Sp ec 0.81–0.84; 8-OHdG in non-DM: AUC 0.84; combinations: AUC up to 0.97–1.00	High-performance multi-marker diagnostic panel for periodontal disease stratified by diabetes status	Strong potential for multiplex salivary diagnostics; requires laboratory ELISA or biosensor development; combinations outperform single biomarkers
Multi-marker panels	MMP-8 + IL-1β	Miller CS et al., 2021	Case-control	29; T2DM-PD 32; T2DM-PH	Saliva	ELISA/multiplex	Discrimination of periodontitis in T2DM	Sens 24.1%; Spec 98%; PPV 87.5%	High specificity adjunct	Low sensitivity; combined with clinical data
	Microbiome + host ratios	Miller CS et al., 2026	Secondary analysis	92; T2DM-PD T2DM-PH	Saliva	Immunoassay + 16S rRNA	Improved disease classification	AUC up to 0.873; Sens 0.893	Enhanced diagnostic precision	Requires multiplex & sequencing
	miRNA panel (146a, 146b, 155, 203)	Al-Rawi et al., 2020	Cross-sectional	53; T2DM-PD, T2DM-PH	Saliva	RT-qPCR	Differential diagnosis DM vs. periodontitis	AUC > 0.9 for miR-146a/b & miR-155 in DM detection; periodontal prediction weaker in DM group (AUC = 0.6–0.7)	Biomarker panel for systemic-oral disease interaction	Good diagnostic accuracy but requires lab infrastructure; limited standalone periodontal prediction in diabetics
Integrated multi-omics biomarkers	Combined salivary bacterial species, 21 salivary metabolites (including lactic acid, glutamic acid, putrescine, glucose, galactose, propionate, and butyrate), and microbial diversity indices.	Diao et al., 2025	Cross-sectional integrated multi-omics study	42; T2DM patients with varying glycemic control status	Saliva, GCF	Full-length 16S rRNA sequencing, LC-MS/metabolomic profiling	Non-invasive differentiation of glycemic control states and periodontal dysbiosis in T2DM	Salivary bacterial panel AUC = 0.9151; salivary metabolite panel AUC = 0.9271; GCF bacterial model AUC = 0.7666	Comprehensive host-microbial-metabolic profiling with strong diagnostic capability for metabolic risk stratification and periodontal disease assessment	Requires advanced sequencing and metabolomic infrastructure, bioinformatics expertise, assay standardization, and external validation before routine clinical implementation

aMMP-8, active matrix metalloproteinase-8; AGE, advanced glycation end products; AUC, area under the receiver operating characteristic curve; BoP, bleeding on probing; T2DM, Type 2 diabetes mellitus; ELISA, enzyme-linked immunosorbent assay; GCF, gingival crevicular fluid; HbA1c, glycated hemoglobin; T2DM-PH, Type 2 Diabetes Mellitus with Periodontal Health; T2DM-PD, Type 2 Diabetes Mellitus with Periodontitis; SH-PH, systemically and periodontally healthy; SH-PD, systemically healthy with periodontitis; hsCRP, high-sensitivity C-reactive protein; IL, interleukin; MDA, malondialdehyde; MetS, metabolic syndrome; miRNA, microRNA; OP, osteoporosis; PD, periodontitis; PoCT/POCT, point-of-care testing; RAGE, receptor for advanced glycation end products; RT-qPCR, reverse transcription quantitative polymerase chain reaction; Spec, specificity; Sens, sensitivity; 4-HNE, 4-hydroxy-2-nonenal; 8-OHdG, 8-hydroxy-2′-deoxyguanosine.

## 3 Results

Consistent with the analytical framework defined *a priori* (Section 2.9), the charted evidence was organized and synthesized according to the distinct clinical objective addressed by each study rather than by biomarker class. Six clinical objectives were distinguished, each interpreted only against its own reference standard: (i) diagnosis of periodontitis (periodontal health vs. periodontitis); (ii) differentiation of diabetic from non-diabetic periodontitis (diabetes or metabolic status within periodontitis); (iii) assessment of periodontal disease severity (continuous or staged periodontal parameters: PPD, CAL, and BoP); (iv) glycemic assessment (HbA1c or glycemic-control category); (v) treatment response and disease monitoring (within-patient baseline vs. post-therapy); and (vi) screening and case-finding in integrated dental-medical care (detection of previously undiagnosed dysglycemia or periodontitis). Diagnostic-accuracy estimates (sensitivity, specificity, and AUC), correlations with periodontal parameters, and associations with glycemic markers represent different outcomes and are therefore reported within, and not across, these objectives. The biological pathways underlying these associations are considered separately in the Discussion. The mechanistic links and diagnostic-performance outcomes charted for each objective are summarized in Tables 3, 4; each biomarker is mapped to its clinical objective and reference standard in Table 2; and a complete study-by-study overview is provided in Supplementary Material S3.

Across the 40 included studies, the evidence base was predominantly cross-sectional, with smaller numbers of case-control and cohort or point-of-care validation studies. Most studies enrolled adults with type 2 diabetes, and a minority also included prediabetes or normoglycemic comparison groups. A complete study-by-study characterization, including country, design, sample size, participant age, diabetes and periodontal case definitions, oral fluid matrix, biomarkers, and assay platform, is provided in Supplementary Material S3.

### 3.1 Diagnosis of periodontitis (reference standard: periodontal health vs. periodontitis)

This objective concerns discrimination of periodontitis from periodontal health. Among individual salivary analytes, which demonstrated moderate-to-good diagnostic performance for periodontitis, aMMP-8 showed good discriminatory ability across oral fluids, with area under the curve (AUCs) of 0.84 (oral rinse), 0.81 (saliva), and 0.75 (GCF) for periodontitis vs. health (24). IL-1β demonstrated consistently high diagnostic accuracy (AUC 0.87–0.93) (25). IL-6 showed moderate performance (AUC 0.79–0.86), whereas salivary IL-6 demonstrated lower sensitivity (53.3%), specificity (68.6%), and accuracy (60%) (26). Among emerging analytes evaluated against the same reference standard, asprosin demonstrated good performance (AUC = 0.884; sensitivity and specificity 81.8%), IL-40 moderate performance (AUC 0.75–0.80), and IL-39 poor discrimination (AUC 0.376–0.461) (25). MMP-8 demonstrated the strongest individual performance among single biomarkers in one cohort (OR = 8.12; sensitivity = 24.1%; specificity = 98%) (14). Salivary miR-155, miR-146a/b, and miR-203 further supported the diagnostic relevance of salivary miRNA profiles (27).

Multi-marker approaches generally reported higher diagnostic accuracy than single biomarkers against this reference standard. Biomarker ratios improved diagnostic performance, with MMP-8/TIMP-1 achieving an AUC of 0.828 and MMP-9/TIMP-1 an AUC of 0.821 (both *p* = 0.0001). Pairwise models such as P. gingivalis + MMP-9/TIMP-1 achieved AUC = 0.874 (accuracy = 0.837), while P. gingivalis + Mycoplasma faucium reached AUC = 0.828. Multimarker logistic regression panels showed the highest overall performance, with 3-marker models achieving AUCs of 0.89–0.93 and 6-marker panels reaching AUCs of 0.95 (28).

### 3.2 Differentiation of diabetic from non-diabetic periodontitis (reference standard: diabetes or metabolic status within periodontitis)

Several studies evaluated whether biomarkers distinguish diabetic (or metabolic) from non-diabetic periodontitis, a reference standard distinct from periodontitis-vs.-health discrimination. For aMMP-8, discrimination between metabolic and non-metabolic periodontitis remained limited, particularly in saliva (AUC = 0.53) and GCF (AUC = 0.43), with modest performance in oral rinse (AUC = 0.68); diagnostic ability was higher for metabolic syndrome-associated periodontitis relative to health (AUCs 0.89, 0.85, and 0.82 for oral rinse, saliva, and GCF, respectively) (24). IL-1β showed excellent discrimination for diabetic periodontitis (AUC 0.872–0.975, *p* < 0.001) (25). Combined oxidative-stress and glycation panels achieved AUCs of 0.92–1.00 in diabetic periodontitis (8-OHdG/4-HNE/AGE/RAGE) compared with 0.79–0.96 in non-diabetic populations [8-OHdG/MDA/high-sensitivity c-reactive protein (hsCRP) panels] (29), and cumulative biomarker-burden approaches (≥3 elevated analytes) improved diabetic periodontitis detection to 55% (14).

Consistent with these accuracy estimates, several analytes were higher in diabetic than in non-diabetic periodontitis. Salivary MMP-8 increased progressively with disease severity, reaching highest levels in diabetic periodontitis, followed by non-diabetic periodontitis and health (*p* < 0.001) (14, 30, 31). MMP-9 was consistently elevated in diabetic periodontitis and strongly correlated with aMMP-8 (*ρ* = 0.721, *p* = 0.019), indicating coordinated collagenolytic and gelatinolytic activity (32, 33). AGE levels increased stepwise with disease severity, peaking in diabetic periodontitis (521.9 pg/mL) compared with non-diabetic periodontitis (234.8 pg/mL) and health (87.2 pg/mL; *p* < 0.01); these gingival crevicular fluid concentrations were measured by ELISA (34). In GCF, 4-HNE showed stronger associations with PPD and CAL in diabetic than in non-diabetic periodontitis, indicating amplified oxidative stress-disease coupling under hyperglycemia (35), and total oxidative stress was highest in diabetic periodontitis (*p* ≤ 0.001) (36). miR-223, miR-200b, and miR-203 were significantly dysregulated in GCF, with greater expression in T2DM-associated periodontitis and accompanied by increased TNF-α and reduced IL-10 (37, 38).

### 3.3 Assessment of periodontal disease severity (reference standard: periodontal parameters—PPD, CAL, BoP, and staging)

Against periodontal clinical parameters, MMP-8 correlated with periodontal indices including PI (plaque index), BoP, PPD, and CAL (*r* = 0.459–0.516; *p* < 0.001), supporting enhanced matrix degradation under hyperglycemia (39). Across oral fluids, aMMP-8 correlated strongly with periodontal parameters in saliva (BoP *r* = 0.601, PPD *r* = 0.705, CAL *r* = 0.776), oral rinse (*r* = 0.559–0.770), and GCF (*r* = 0.546–0.743), with saliva showing the strongest associations (24); aMMP-8 was also correlated with neutrophil elastase (*ρ* = 0.729), MMP-9 (*ρ* = 0.721), and the MMP-8/TIMP-1 ratio (*ρ* = 0.879; all *p* < 0.05) (33). MMP-2 activity increased with disease severity (*p* < 0.05), whereas TIMP-1, although elevated in periodontitis, was reduced under poor glycemic control, resulting in increased MMP-8/TIMP-1 ratios and impaired inhibition of proteolysis (12, 40). *β*-glucuronidase levels were higher in periodontitis (*β* = 0.75, *p* < 0.001), with a smaller independent effect of T2DM (*β* = 0.41, *p* < 0.001); overall levels were 0.4 log units higher in diabetics, but periodontal status remained the dominant determinant of *β*-glucuronidase and IL-1β expression (41).

Among cytokines, IL-1β was consistently elevated in diabetic periodontitis (3.82 ± 1.76 vs. 2.35 ± 1.21 pg/mL; *p* = 0.0005) and strongly correlated with periodontal parameters including PPD and BoP (*r* = 0.49–0.71, *p* < 0.0001) (12, 14, 25); within this objective it remained the strongest cytokine predictor of periodontal destruction (CAL *r* = 0.611; PPD *r* = 0.499; PI *r* = 0.585; *p* < 0.01), outperforming IL-40, asprosin, and IL-39 (25). Regression analysis indicated a stronger association of IL-1β with periodontal inflammation than with diabetes alone (*β* = 2.037 vs. 0.497) (39, 41). IL-6 correlated with periodontal severity (*r* = 0.18–0.32), although weaker correlations with clinical indices suggested a more modulatory role (14, 26, 42). IL-17 was positively associated with disease severity, whereas Del-1 showed inverse correlations with periodontal parameters (*r* = −0.57 to −0.66) (43, 44). Elevated IL-18, absent in melanoma 2 (AIM2), and interferon gamma inducible protein 16 (IFI16) correlated strongly with periodontal indices (*r* = 0.458–0.895), and IL-34 and colony stimulating factor-1 (CSF-1) also correlated with periodontal measures (*r* = 0.357–0.657) (45, 46). Additional mediators, including macrophage inflammatory protein-1 alpha (MIP-1*α*), interferon-alpha (IFN-α), and B-cell Activating Factor (BAFF), showed stronger associations with microbial dysbiosis and disease severity in diabetic disease (47).

Glycation and oxidative markers likewise tracked periodontal severity. Oxidative-stress biomarkers were consistently elevated in periodontitis and further increased in diabetic periodontitis, with increased lipid peroxidation products [malondialdehyde (MDA) and 4-hydroxynonenal (4-HNE)] and reduced antioxidant defenses such as superoxide dismutase (SOD), catalase, and glutathione reductase (29, 35, 48, 49). AGE levels correlated positively with PPD and bone loss, with combined models explaining up to 59% of variance (R² = 0.59), and in PISF, AGE levels correlated positively with probing depth (PD) (*p* = 0.0221–0.0371) and marginal bone loss (MBL) (*p* = 0.0117–0.0425), while showing an inverse association with PI (*p* = 0.0276) (50, 51). In GCF, periodontal severity (PPD ≥4 mm) correlated negatively with C-peptide (*r* = −0.240, *p* < 0.01) and positively with gastric inhibitory polypeptide (GIP) (*r* = 0.255, *p* < 0.01) and visfatin (*r* = 0.241, *p* < 0.01), indicating altered insulin-regulatory and adipokine signaling (52). The AGE/sRAGE imbalance also correlated with periodontal parameters (53, 54). Salivary 8-hydroxy-2′-deoxyguanosine (8-OHdG) and MDA correlated positively with BoP and CAL (*r* = 0.48–0.49; *p* < 0.001) and inversely with antioxidant capacity (29, 49), and sulfiredoxin correlated strongly with total oxidant status (TOS) (*r* = 0.65, *p* ≤ 0.005) (36). Salivary 8-Isoprostane (8-IsoP) showed significant positive correlations with gingival index (GI), PPD, and CAL loss in periodontitis patients with and without diabetes (*r* = 0.70–0.99; *P* < 0.001) (55). Among epigenetic markers, miR-223 correlated with CAL (*r* = 0.322), PPD (*r* = 0.203), and TNF-α (*r* = 0.229), while miR-200b correlated with CAL (*r* = 0.233) and PPD (*r* = 0.220) (37).

### 3.4 Glycemic assessment (reference standard: HbA1c or glycemic-control category)

For association with glycemic status, salivary RAGE was the only independent predictor of HbA1c (*β* = 0.161, *p* < 0.001; R² = 0.509), whereas AGE, sRAGE, and aMMP-8 were not significant predictors of glycemic status (54). MMP-8 correlated with HbA1c (*r* = 0.409) (39), and aMMP-8 correlated with prediabetes in severe periodontitis (*ρ* = 0.646, *p* = 0.044) (33). Glycated albumin was elevated in diabetes and correlated with HbA1c (56), and the AGE/sRAGE imbalance (increased AGE, reduced sRAGE) correlated with HbA1c (53, 54). In PISF, AGE levels increased with worsening glycemic status (50, 51). Among cytokines, IL-6 was associated with HbA1c (*r* = 0.60, *p* < 0.01) (14, 26, 42), IL-17 with HbA1c (*r* = 0.329–0.645) (43, 44), and IL-18 with glycemic markers (*r* = 0.280–0.331; *p* ≤ 0.017) (57); IL-18/AIM2/IFI16 (*r* = 0.465–0.788; *p* < 0.01) and IL-34/CSF-1 (*r* = 0.588–0.676; *p* < 0.01) also correlated with HbA1c (45, 46). Salivary IL-1β and soluble triggering receptor expressed on myeloid cells-1 (sTREM-1) correlated with both periodontal indices and HbA1c (*r* = 0.412–0.688; *p* < 0.001) (39, 41). Nitric oxide levels were elevated in poorly controlled diabetes (*p* < 0.05) (40), and HbA1c was a significant predictor of sulfiredoxin expression (*p* ≤ 0.008) (36). Glycemic disturbance was also associated with significant microbial-metabolite correlations: Neisseria elongata correlated negatively with putrescine, lactic acid, and 3-phenyllactic acid; 4-hydroxyphenylacetic acid correlated positively with periodontitis-associated species; and niacinamide correlated negatively with Veillonella atypica and Prevotella pallens (*p* < 0.05), indicating glycemia-associated coupling between oral microbial composition and metabolite profiles (58).

For discrimination of glycemic-control states, a bacterial biomarker panel comprising nine salivary species discriminated glycemic control from impaired glycemic control (AUC = 0.9151), while the corresponding GCF-based model showed moderate accuracy (AUC = 0.7666); a salivary metabolite-based model incorporating 21 key metabolites achieved excellent performance (AUC = 0.9271) (58).

### 3.5 Treatment response and disease monitoring (reference standard: within-patient baseline vs. post-therapy)

Because studies focusing on the effects of therapeutic interventions were excluded *a priori* (Section 2.5), evidence charted under this objective was limited. No included study reported longitudinal within-patient biomarker change against a baseline-vs.-post-therapy reference standard as a primary charted outcome; the responsiveness of aMMP-8 to periodontal therapy is therefore considered in the Discussion on the basis of the broader literature rather than the charted evidence, and this gap is identified as a priority for future work.

### 3.6 Screening and case-finding in integrated dental-medical care (reference standard: detection of previously undiagnosed dysglycemia or periodontitis)

Within the screening objective, biomarker performance was evaluated for its incremental value in identifying previously undiagnosed dysglycemia or periodontitis when combined with clinical risk factors. Integration of aMMP-8 with age ≥45 years and body mass index (BMI) improved diagnostic accuracy from AUC 0.683 to 0.759 (*p* = 0.001), supporting its utility in risk-based screening (17). In chair-side point-of-care testing, inclusion of aMMP-8 improved model performance from AUC 0.651 (age + BMI model) to 0.660, further increasing to 0.711 with the addition of periodontitis stage and to 0.713 in the full combined model (age, BMI, aMMP-8, and periodontal stage) (17). These findings indicate that although aMMP-8 alone has limited discriminatory ability for this objective, multimodal models substantially improve performance for diabetic periodontitis and dysglycemia screening.

## 4 Discussion

The principal contribution of this review is not a further catalog of biomarkers but an explicit organization of the evidence along a biological-to-clinical translation axis, coupled with a point-of-care readiness appraisal (Table 1). This focus distinguishes it from prior mechanistic and narrative reviews of the periodontitis and diabetes relationship (11, 13) and from biomarker-specific systematic reviews (59), which did not appraise readiness for chair-side application.

### 4.1 Biological significance

#### 4.1.1 Proteolytic and matrix degradation pathways

Dysregulated MMP activity, particularly aMMP-8, is central to tissue destruction in diabetic periodontitis. In T2DM, hyperglycemia-driven chronic inflammation enhances neutrophil recruitment and release of latent MMP-8, which is activated through independent and/or cooperative action of oxidative stress and proteolytic enzymes. Salivary MMP-8 and its active form (aMMP-8) increase progressively from health to periodontitis and are further amplified in the presence of diabetes, reflecting the synergistic effect of hyperglycemia and local inflammation (9, 14, 30, 31, 39, 60).

Hyperglycemia promotes chronic low-grade inflammation and neutrophil priming, enhancing the release of latent MMP-8, which is subsequently activated by oxidative stress and proteolytic enzymes. Poor glycemic control (HbA1c >8%) correlates with *Prevotella/Porphyromonas* dominance and short-chain fatty acid depletion, amplifying AGE production and RAGE signaling. Advanced glycation end products and ROS further upregulate MMP expression via pro-inflammatory cytokines such as IL-1β and TNF-α, shifting the balance toward collagen degradation. Strong correlations were observed between aMMP-8 and inflammatory mediators, MMP-9 and polymorphonuclear elastases, and with clinical parameters such as PPD and BoP (24, 33).

Concurrently, reduced TIMP-1 activity contributes to a protease-antiprotease imbalance, reflected in the elevated MMP-8/TIMP-1 ratio observed in severe disease (12, 40). In contrast, Peniche-Palma et al. (2019) reported no consistent differences in MMP-9 and myeloperoxidase (MPO) across glycemic or periodontal status groups, suggesting that unlike aMMP-8, these markers may not reliably capture combined metabolic-inflammatory effects (61).

MMP-9, although less collagen-specific, is also significantly elevated and correlates with aMMP-8, indicating coordinated activation of collagenolytic and gelatinolytic pathways (15, 17, 32–34, 60). AGE-mediated collagen crosslinking further increases matrix susceptibility to degradation and enhances MMP expression via RAGE- nuclear factor kappa-light-chain-enhancer of activated B cells (NF-*Κ*b) signaling (34). Clinically, aMMP-8 emerges as a sensitive marker of active tissue destruction, with levels >20 ng/mL indicating ongoing collagen degradation. Its strong association with periodontal parameters and its responsiveness to periodontal therapy highlight its value as a dynamic biomarker of disease activity (15, 17).

#### 4.1.2 Cytokine-Driven immune amplification

Cytokine profiling in diabetic periodontitis demonstrates synergistic inflammatory amplification beyond additive effects of either condition alone. Elevated IL-1β, IL-6, tumor necrosis factor-alpha (TNF-α), and IL-17, together with reduced IL-10, indicate profound immune dysregulation (12, 14, 25). This convergence of systemic and local inflammation creates a self-sustaining inflammatory loop that accelerates both tissue destruction and metabolic dysregulation.

IL-6 links metabolic and periodontal inflammation, correlating with HbA1c while showing weaker associations with periodontal parameters, supporting a modulatory rather than primary effector role (26, 42). The markedly elevated IL-1β/IL-10 ratio highlights loss of immunoregulatory control, with IL-1β acting as a key upstream mediator of MMP activation and osteoclastogenesis and salivary IL-1β associations with periodontal parameters independent of diabetes further reinforce its central role (41).

IL-17 contributes to neutrophil recruitment and bone resorption, while Del-1 inversely correlates with periodontal and metabolic parameters, reflecting an IL-17/Del-1 imbalance in sustained inflammation (43, 44). Additional mediators, including MIP-1*α*, BAFF, IL-18, AIM2, IFI16, IL-34, and colony stimulating factor-1 (CSF-1), show stronger associations with dysbiosis and disease severity in diabetic periodontitis (45–47). Collectively, these findings support a coordinated host-microbial inflammatory network driving periodontal destruction in diabetes.

#### 4.1.3 Metabolic-Inflammatory interfaces

The AGE-RAGE axis represents a key mechanistic link between hyperglycemia and periodontal destruction. AGE and RAGE levels show strong associations with both glycemic control and periodontal parameters (34, 62). AGE accumulation in GCF and PISF correlates with PPD, CAL, and bone loss, reinforcing its role in inflammatory tissue breakdown.

This axis is reinforced by adipokine imbalance (↑resistin, ↓adiponectin), sustaining a chronic metabolic-inflammatory loop. AGE levels also correlate with implant-related bone loss and inversely with plaque index, indicating disease severity rather than biofilm quantity alone (50, 51).

T2DM participants showed significantly higher HbA1c and AGE levels, with LL-37 positively correlating with both markers, whereas non-diabetic individuals demonstrated weaker and less consistent associations. These findings suggest a stronger glycation-innate immunity interaction in diabetic periodontitis (63).

Salivary RAGE has been identified as an independent predictor of glycemic status, further supporting its role as a systemic-local interface biomarker (54). Reduced soluble RAGE (sRAGE) levels in diabetic periodontitis remove a protective decoy mechanism, resulting in an elevated AGE/sRAGE ratio and enhanced inflammatory signaling (53). Furthermore, lower C-peptide levels and higher glucose-dependent insulinotropic polypeptide (GIP) and visfatin levels were observed, reflecting a coordinated disruption of insulin regulation alongside enhanced adipose-derived inflammatory signaling in periodontitis (52). Together, these findings highlight a bidirectional relationship in which hyperglycemia exacerbates periodontal inflammation, while periodontal inflammation contributes to systemic metabolic dysregulation.

#### 4.1.4 Oxidative stress and innate immune dysregulation

Oxidative stress is significantly elevated in periodontitis and further amplified in diabetes, as demonstrated by the results and supported by studies on salivary 8-OHdG, MDA, and 4-HNE. Overall, evidence indicates a consistent oxidative imbalance in periodontitis that is significantly exacerbated by diabetes and closely linked to clinical periodontal destruction (29, 35, 49). Salivary 8-IsoP is strongly associated with periodontal inflammation and tissue destruction, indicating increased oxidative stress with disease severity, while diabetes does not appear to independently influence its levels (55).

These markers show strong positive correlations with BoP and CAL, indicating their role in tissue damage. Neutrophil hyperactivation plays a key role in this process, with elevated levels of MPO, calprotectin (S100A8/A9), and related mediators contributing to excessive ROS production and tissue destruction. This reflects a shift from protective to destructive innate immune responses under hyperglycemic conditions. *P. gingivalis*-derived gingipains directly activate neutrophils, elevating MPO/calprotectin and ROS that degrade collagen independently of host MMPs (28). This shifts innate immunity from protection to pathology under hyperglycemia.

#### 4.1.5 Epigenetic regulation via MicroRNAs

Epigenetic mechanisms, particularly miRNAs, add an additional layer of regulation to diabetic periodontitis. miRNAs such as miR-223 and miR-200b are upregulated and show strong correlations with periodontal parameters and inflammatory cytokines (59). Importantly, salivary miRNAs, including miR-146a and miR-155, demonstrate strong diagnostic potential, with high accuracy in distinguishing disease states. miR-146a shows diagnostic accuracy exceeding 85%, while miR-155 and related markers reflect dysregulated inflammatory signaling pathways (27). These findings suggest that miRNAs may serve as upstream regulators and potential therapeutic targets, although their clinical application requires further validation.

#### 4.1.6 Stratification of the evidence by clinical objective

The reviewed evidence addressed several distinct clinical objectives that are not directly interchangeable: discrimination of periodontitis from periodontal health (for example, aMMP-8 and IL-1β), discrimination of diabetic from non-diabetic periodontitis (for example, oxidative-stress and glycation panels), assessment of glycemic control or dysglycemia risk (for example, salivary RAGE and microbiome-to-metabolome panels), grading of periodontal severity, and monitoring of treatment response (for example, aMMP-8 after therapy). Because diagnostic estimates derived for one objective do not transfer to another, and because periodontal and diabetes case definitions varied across studies, biomarker performance is reported within, rather than across, these objectives. Accordingly, the Results (Section 3) are organized by these clinical objectives, and each biomarker is mapped to its objective and reference standard in Table 2.

Beyond aMMP-8, several biomarker classes show complementary and sometimes comparable value: salivary IL-1β achieved among the highest single-analyte accuracy; glycation markers (AGE, RAGE, and sRAGE) provided the closest link to glycemic status; oxidative-stress markers (8-OHdG, 4-HNE, and MDA) tracked periodontal destruction under hyperglycemia; and metabolomic and microRNA signatures showed high discrimination in early multi-omics work, albeit with greater platform and validation requirements. A balanced translational appraisal therefore treats aMMP-8 as the most operationally mature option rather than the analytically superior one.

### 4.2 Clinical translation readiness

Chair-side aMMP-8 POCT enables rapid, non-invasive detection and monitoring of periodontal inflammation in T2DM populations, demonstrating clinically useful but study-dependent diagnostic accuracy that varies with study population, reference standard, and cut-off threshold and significant associations with HbA1c and treatment response (15, 54, 64). In combination with clinical risk factors, aMMP-8 testing also improves identification of previously undiagnosed dysglycemia (17).

Among individual biomarkers, IL-1β and aMMP-8 demonstrate the strongest diagnostic performance (AUCs approximately 0.82–0.93), reflecting their central roles in inflammatory signaling and collagen degradation (14, 25). aMMP-8 is particularly valuable because it reflects active tissue destruction rather than cumulative periodontal damage. Its diagnostic performance further improves when combined with systemic and clinical parameters in multivariate models (17, 65). These comparisons refer specifically to the diagnosis of periodontitis (periodontal health vs. periodontitis) and are not extended to other clinical objectives, each of which has a different reference standard.

Other biomarkers, including IL-6, oxidative stress markers, and glycation-related molecules, show moderate performance individually but provide complementary information. Oxidative stress biomarkers such as 8-OHdG and 4-HNE demonstrate strong diagnostic accuracy in specific populations, particularly when combined (29). Overall, multimarker approaches generally reported higher accuracy than individual biomarkers, although estimates were not pooled or quality graded. Panels incorporating combinations such as 8-OHdG/4-HNE/AGE/RAGE achieve excellent discriminatory performance in diabetic populations (AUCs up to 0.92–1.00), while broader multimarker models report AUC values approaching 0.95 (28, 29).

In line with emerging multi-omics approaches, Diao et al. (2025) showed that integrated salivary microbiome and metabolome profiling provides strong diagnostic accuracy for distinguishing glycemic control states in T2DM-associated periodontal conditions, with higher reported performance of salivary bacterial and metabolite panels than GCF-based models, supporting the value of multi-marker oral fluid approaches for non-invasive metabolic risk stratification (58).

To make the notions of clinical utility and chair-side readiness explicit, each biomarker class was appraised against six translational criteria adapted from biomarker-development frameworks: (1) analytical validity (assay accuracy, precision, and standardization); (2) diagnostic validity (sensitivity, specificity, and AUC against a defined reference standard); (3) external validation (independent replication across populations); (4) feasibility of chair-side or point-of-care implementation; (5) cost-effectiveness; and (6) regulatory approval and integration into care pathways. Table 1 maps the principal biomarkers against these criteria. Within this framework, aMMP-8 point-of-care testing is currently the only option meeting most criteria, whereas cytokine, glycation, oxidative-stress, metabolomic, and microRNA markers remain at the analytical or diagnostic-validity stage and require external validation before implementation. Because readiness depends on the clinical objective, each biomarker class is appraised for the objective it primarily serves and against the corresponding reference standard rather than for clinical implementation in general; Table 1 records this primary objective for each class, and Table 2 details the objective-to-reference-standard mapping.

### 4.3 Chair-side implementation and integrated care pathways

Point-of-care testing (POCT), particularly aMMP-8-based assays, represents a practical strategy for integrating oral biomarker assessment into routine periodontal and diabetes care. Chair-side assays enable rapid, non-invasive evaluation of periodontal inflammation with clinically useful diagnostic performance, typically demonstrating sensitivity and specificity exceeding 75%–85% (15, 17, 33). Current evidence also indicates that periodontal inflammatory burden in individuals with diabetes and prediabetes can be effectively monitored using aMMP-8 POCT.

Importantly, non-surgical periodontal therapy has been shown to improve glycemic control, supporting the rationale for early periodontal intervention in diabetes management (4). Correspondingly, aMMP-8 levels decrease following periodontal therapy and correlate with changes in glycemic status, supporting their utility for dynamic monitoring of both periodontal and metabolic outcomes. Western immunoblot analyses further confirm elevated activated MMP-8 in oral rinse samples from diseased individuals, while healthy controls demonstrate minimal or absent MMP immunoreactivity (17).

Emerging technologies such as ORALyzer further expand chair-side capabilities through rapid multiplex detection of inflammatory mediators including IL-1β, aMMP-8, and IL-6. These systems demonstrate strong agreement with ELISA-based measurements (*r* = 0.89–0.94) and comparable diagnostic accuracy (AUC 0.84–0.88), supporting integrated chair-side assessment of both proteolytic activity and inflammatory burden.

POCT platforms may also facilitate bidirectional screening within integrated care pathways. Dental settings may help identify individuals at risk of undiagnosed dysglycemia, whereas medical settings may assist in detecting subclinical periodontal inflammation in patients with diabetes. In this context, aMMP-8 testing functions as a translational bridge between dentistry and medicine, aligning with emerging guideline-driven integration of oral and systemic healthcare.

Additional support for this integrated approach comes from evidence that periodontal therapy improves glycemic control, together with the anti-inflammatory effects of metabolic therapies such as glucagon-like peptide-1 receptor agonists and dipeptidyl peptidase-4 inhibitors (66–68). A streamlined implementation pathway incorporating brief risk assessment, chair-side examination, and POCT-based stratification may enable immediate risk categorization, referral, and follow-up. Integration into electronic health records could further support continuous bidirectional communication between dental and medical providers. Nevertheless, widespread implementation will require assay standardization, validation of cost-effectiveness, reimbursement integration, and harmonization within primary healthcare systems. Because most supporting studies were cross-sectional, claims regarding monitoring, prediction, and integrated implementation should be regarded as hypotheses for prospective testing rather than established practice.

Because this review is framed within cardiometabolic health, the oral biomarkers mapped here are relevant beyond the periodontal-to-glycemic axis. Several of them, notably IL-6, IL-1β, high-sensitivity C-reactive protein, and MMP-8, are also recognized markers of systemic inflammation and vascular risk, and the 2024 EFP and WONCA Europe consensus explicitly positions periodontitis alongside cardiovascular disease as a comorbidity of diabetes (3). Periodontal inflammation contributes to the systemic inflammatory burden implicated in atherogenesis, and modern antidiabetic therapies with cardiovascular benefit, such as glucagon-like peptide-1 receptor agonists and dipeptidyl peptidase-4 inhibitors, also exert anti-inflammatory effects relevant to the periodontium (68). Oral-fluid biomarker testing could therefore contribute to integrated cardiometabolic risk assessment in type 2 diabetes, although direct evidence linking oral-fluid biomarker levels to cardiovascular outcomes in this population remains limited.

### 4.4 Research gaps and priorities for future work

Despite progress in oral biomarker research for diabetic periodontitis, clinical translation faces key barriers: lack of standardization in sample collection/analysis, heterogeneous cross-sectional studies that limit temporal/predictive validity, and insufficient integration of guidelines. Emerging multi-omics approaches lack external validation and scalability. Future priorities include longitudinal RCTs, standardized cut-offs, cost-effectiveness analyses, population validation, and implementation research addressing workflow, reimbursement (cited by 71% of surveyed dentists), time constraints (82%), and limited familiarity with biomarker interpretation and harmonized dental-medical protocols (18).

#### 4.4.1 Methodological considerations and study quality

Because scoping reviews map rather than appraise evidence, study quality, certainty, and risk of bias were not formally assessed. The included studies varied in sample size, adjustment for confounders, assay validation, and case definitions, and the diagnostic-accuracy estimates summarized here were drawn from studies with differing designs, populations, reference standards, and cut-offs. Reported sensitivity, specificity, odds ratios, and AUC values are therefore presented as a descriptive map and should not be interpreted as pooled or directly comparable performance metrics. Where single studies reported unusually high estimates, for example a specificity approaching 100 percent, these reflect specific populations and thresholds and require external validation.

#### 4.4.2 Strengths

This review has several strengths. The search spanned five major databases and was supplemented by grey-literature sources, forward and backward citation tracking, and hand searching of leading periodontology journals. The methodology followed JBI scoping-review guidance and PRISMA-ScR reporting, with dual independent screening and charting. The biological-to-clinical translation framework and the accompanying point-of-care readiness appraisal (Table 1) provide a structured map intended to be directly useful to clinicians and to investigators planning confirmatory studies.

### 4.5 Limitations

Several limitations should be acknowledged. First, no priori protocol was registered with the Open Science Framework or PROSPERO, which may increase the risk of selection bias despite dual independent screening. Second, grey-literature research was limited to the first 200 Google Scholar results, ClinicalTrials.gov, and the WHO ICTRP, potentially missing unpublished or regionally indexed studies. Third, substantial heterogeneity in periodontal definitions, biomarker assays, oral fluid matrices, and statistical methods limited cross-study comparability and precluded meta-analysis. Fourth, most included studies were cross-sectional, limiting causal inference and assessment of longitudinal biomarker performance. Finally, in accordance with JBI scoping review methodology, no formal risk-of-bias assessment was conducted. In addition, the low number of database records retrieved (178 before deduplication) may indicate that the search strategy, although specific, was not maximally sensitive; a broader strategy could identify further studies and could, in principle, modify the evidence map. The predominance of cross-sectional designs further limits inferences about prediction, monitoring, and causal direction.

## 5 Conclusion

Oral fluid biomarkers demonstrate robust multi-axis involvement in diabetic periodontitis, spanning proteolytic, inflammatory, metabolic, oxidative, and epigenetic pathways. Among the biomarkers mapped, aMMP-8 currently has the most extensive point-of-care validation, and multi-marker panels generally report higher diagnostic accuracy than single analytes. Because this is a scoping review without formal quality appraisal or quantitative synthesis, these observations describe the distribution of available evidence rather than a graded ranking of biomarker superiority. Integration of chair-side POCT with systemic screening represents a feasible pathway for early detection and integrated periodontal-metabolic care. IL-1β and selected microRNAs showed comparable individual diagnostic performance but currently require laboratory platforms. Multi-marker panels consistently outperformed single biomarkers, in diabetic periodontitis cohorts, and bidirectional dental-medical screening appears feasible when aMMP-8 chair-side testing is combined with HbA1c point-of-care measurement. Clinical adoption, however, remains constrained by assay heterogeneity, the absence of standardized cut-offs, limited longitudinal evidence, and gaps in reimbursement and interdisciplinary workflow. Priorities for future work are standardized assay protocols, prospective multi-center validation of multi-marker panels, and implementation studies that integrate oral fluid testing into diabetes and periodontal care pathways. These observations hold within, and are not transferred across, the clinical objectives defined in Section 2.9, each interpreted against its own reference standard.
