# Supplementary material for: From biological evidence to clinical application: a scoping review of oral fluid biomarkers in type 2 diabetic periodontitis
Source: Front Oral Health. 2026 Jul 28;7:1892626. doi: 10.3389/froh.2026.1892626 (PMC13457215; doi:10.3389/froh.2026.1892626)
Supplement: Supplementary file 1 [file Table1.docx]

**Supplementary Material S1. PRISMA-ScR Checklist**

| **SECTION / TOPIC** | **#** | **CHECKLIST ITEM** | **REPORTED ON (Section / Page)** |
| --- | --- | --- | --- |
| Title | 1 | Identify the report as a scoping review. | Title page (page 1) |
| Abstract | 2 | Provide a structured summary that includes (as applicable) background, objectives, eligibility criteria, sources of evidence, charting methods, results, and conclusions that relate to the review questions and objectives. | Abstract (page 2) |
| Rationale | 3 | Describe the rationale for the review in the context of what is already known. | Introduction (page 3) |
| Objectives | 4 | Provide an explicit statement of the questions and objectives being addressed with reference to their key elements (e.g., population or participants, concepts, and context) or other relevant key elements used to conceptualize the review questions and objectives. | Introduction — PCC framework (page 4) |
| Protocol and registration | 5 | Indicate whether a review protocol exists; state if and where it can be accessed (e.g., a Web address); and if available, provide registration information, including the registration number. | Methods section 2.1 — no protocol registered; acknowledged in Limitations (page 13) |
| Eligibility criteria | 6 | Specify characteristics of the sources of evidence used as eligibility criteria (e.g., years considered, language, and publication status), and provide a rationale. | Methods section 2.2 — PCC-based eligibility (page 4) |
| Information sources | 7 | Describe all information sources in the search (e.g., databases with dates of coverage and contact with authors to identify additional sources), as well as the date the most recent search was executed. | Methods section 2.3 — PubMed, Embase, Scopus, Web of Science, Cochrane Library + grey literature (page 4) |
| Search | 8 | Present the full electronic search strategy for at least 1 database, including any limits used, such that it could be repeated. | Methods section 2.3 and Supplementary Material S2 — full PubMed strategy provided |
| Selection of sources of evidence | 9 | State the process for selecting sources of evidence (i.e., screening and eligibility) included in the scoping review. | Methods section 2.5 — dual independent screening with third-reviewer adjudication (page 5) |
| Data charting process | 10 | Describe the methods of charting data from the included sources of evidence (e.g., calibrated forms or forms that have been tested by the team before their use, and whether data charting was done independently or in duplicate) and any processes for obtaining and confirming data from investigators. | Methods section 2.7 (page 5) |
| Data items | 11 | List and define all variables for which data were sought and any assumptions and simplifications made. | Methods section 2.5 and Supplementary Material S3 (page 5) |
| Critical appraisal of individual sources of evidence | 12 | If done, provide a rationale for conducting a critical appraisal of included sources of evidence; describe the methods used and how this information was used in any data synthesis (if appropriate). | Methods section 2.6 — formal risk-of-bias appraisal not performed per JBI scoping-review methodology; noted narratively |
| Synthesis of results | 13 | Describe the methods of handling and summarizing the data that were charted. | Methods section 2.7 — narrative synthesis grouped by biomarker family (CP4) |
| Selection of sources of evidence | 14 | Give numbers of sources of evidence screened, assessed for eligibility, and included in the review, with reasons for exclusions at each stage, ideally using a flow diagram. | Results section 3.1 and Figure 2 (PRISMA-ScR flow) |
| Characteristics of sources of evidence | 15 | For each source of evidence, present characteristics for which data were charted and provide the citations. | Supplementary Material S3 |
| Critical appraisal within sources of evidence | 16 | If done, present data on critical appraisal of included sources of evidence (see item 12). | Not applicable — formal appraisal not performed (see item 12) |
| Results of individual sources of evidence | 17 | For each included source of evidence, present the relevant data that were charted that relate to the review questions and objectives. | Results section 3.2–3.7 — biomarker-family narrative; Tables 2–3 |
| Synthesis of results | 18 | Summarize and/or present the charting results as they relate to the review questions and objectives. | Results section 3.8 and Figure 1 (pathophysiology) |
| Summary of evidence | 19 | Summarize the main results (including an overview of concepts, themes, and types of evidence available), link to the review questions and objectives, and consider the relevance to key groups. | Discussion sections 4.1–4.4 (page 9-13) |
| Limitations | 20 | Discuss the limitations of the scoping review process. | Discussion section 4.5 (page 13) |
| Conclusions | 21 | Provide a general interpretation of the results with respect to the review questions and objectives, as well as potential implications and/or next steps. | Conclusions (page 13) |
| Funding | 22 | Describe sources of funding for the included sources of evidence, as well as sources of funding for the scoping review. Describe the role of the funders of the scoping review. | page 14 |

Checklist based on Tricco, A. C., Lillie, E., Zarin, W., O’Brien, K. K., Colquhoun, H., Levac, D., Moher, D., Peters, M. D. J., Horsley, T., Weeks, L., Hempel, S., Akl, E. A., Chang, C., McGowan, J., Stewart, L., Hartling, L., Aldcroft, A., Wilson, M. G., Garritty, C., … Straus, S. E. (2018). PRISMA extension for scoping reviews (PRISMA-ScR): Checklist and explanation. Annals of Internal Medicine, 169(7), 467–473. https://doi.org/10.7326/M18-0850
