# Supplementary material for: From biological evidence to clinical application: a scoping review of oral fluid biomarkers in type 2 diabetic periodontitis
Source: Front Oral Health. 2026 Jul 28;7:1892626. doi: 10.3389/froh.2026.1892626 (PMC13457215; doi:10.3389/froh.2026.1892626)
Supplement: Supplementary file 2 [file Table2.docx]

**Supplementary Material S2. Database Search Strategies**

Searches were executed on 22 April 2026. Date range: 1990-01-01 to 2026-04-30. Language: no restriction applied at database level; non-English titles were screened with assistance from native-speaker reviewers or translated abstracts. No filter for study design was applied at the database stage.

## 1. PubMed / MEDLINE

Hits: [TBD]. Block structure: Population AND Concept (biomarkers) AND Concept (oral fluids), limited by publication date.

((("Diabetes Mellitus, Type 2"[Mesh]) OR ("Diabetes Mellitus"[Mesh]) OR "type 2 diabetes"[tiab] OR "T2DM"[tiab] OR "diabetes mellitus"[tiab] OR "hyperglycemia"[tiab] OR "hyperglycaemia"[tiab]) AND (("Periodontitis"[Mesh]) OR ("Periodontal Diseases"[Mesh]) OR "periodontitis"[tiab] OR "periodontal disease"[tiab] OR "periodontal inflammation"[tiab])) AND (("Biomarkers"[Mesh]) OR "biomarker*"[tiab] OR "matrix metalloproteinase*"[tiab] OR "MMP-8"[tiab] OR "aMMP-8"[tiab] OR "MMP-9"[tiab] OR "interleukin*"[tiab] OR "IL-1beta"[tiab] OR "IL-6"[tiab] OR "advanced glycation end product*"[tiab] OR "AGE"[tiab] OR "RAGE"[tiab] OR "8-OHdG"[tiab] OR "4-HNE"[tiab] OR "malondialdehyde"[tiab] OR "microRNA*"[tiab] OR "miRNA*"[tiab]) AND (("Saliva"[Mesh]) OR ("Gingival Crevicular Fluid"[Mesh]) OR "saliva*"[tiab] OR "salivary"[tiab] OR "gingival crevicular fluid"[tiab] OR "GCF"[tiab] OR "oral fluid*"[tiab]) AND ("1990"[dp] : "2026"[dp])

## 2. Embase (Elsevier)

Hits: [TBD]. Emtree exploded terms combined with free-text keywords (.ti,ab,kw.).

('diabetes mellitus'/exp OR 'non insulin dependent diabetes mellitus'/exp OR 'type 2 diabetes':ti,ab,kw OR 'diabetes mellitus':ti,ab,kw OR hyperglyc?emia:ti,ab,kw)

AND ('periodontitis'/exp OR 'periodontal disease'/exp OR periodontitis:ti,ab,kw OR 'periodontal disease':ti,ab,kw)

AND ('biological marker'/exp OR biomarker*:ti,ab,kw OR 'matrix metalloproteinase 8':ti,ab,kw OR 'mmp 8':ti,ab,kw OR 'amm p 8':ti,ab,kw OR 'mmp 9':ti,ab,kw OR interleukin*:ti,ab,kw OR 'advanced glycation end product*':ti,ab,kw OR rage:ti,ab,kw OR '8 hydroxy 2 deoxyguanosine':ti,ab,kw OR malondialdehyde:ti,ab,kw OR 'micro rna*':ti,ab,kw)

AND ('saliva'/exp OR 'gingival crevicular fluid'/exp OR saliva*:ti,ab,kw OR salivary:ti,ab,kw OR 'gingival crevicular fluid':ti,ab,kw OR gcf:ti,ab,kw)

AND [1990-2026]/py

## 3. Scopus

Hits: [TBD]. TITLE-ABS-KEY fields across all four PCC blocks.

TITLE-ABS-KEY(("type 2 diabetes" OR "diabetes mellitus" OR hyperglycemia OR hyperglycaemia))

AND TITLE-ABS-KEY((periodontitis OR "periodontal disease"))

AND TITLE-ABS-KEY((biomarker* OR "MMP-8" OR "aMMP-8" OR "MMP-9" OR interleukin* OR "IL-1β" OR "IL-6" OR "AGE" OR "RAGE" OR "8-OHdG" OR "4-HNE" OR malondialdehyde OR microRNA* OR miRNA*))

AND TITLE-ABS-KEY((saliva OR salivary OR "gingival crevicular fluid" OR GCF OR "oral fluid"))

AND PUBYEAR > 1989 AND PUBYEAR < 2027

## 4. Web of Science Core Collection

Hits: [TBD]. TS (topic) field across all four PCC blocks; timespan filter applied after combination.

TS=("type 2 diabetes" OR "diabetes mellitus" OR hyperglycemia OR hyperglycaemia)

AND TS=(periodontitis OR "periodontal disease")

AND TS=(biomarker* OR "MMP-8" OR "aMMP-8" OR "MMP-9" OR interleukin* OR "IL-1β" OR "IL-6" OR AGE OR RAGE OR "8-OHdG" OR "4-HNE" OR malondialdehyde OR microRNA* OR miRNA*)

AND TS=(saliva OR salivary OR "gingival crevicular fluid" OR GCF OR "oral fluid")

Timespan: 1990-2026

## 5. Cochrane Library (CENTRAL and CDSR)

Hits: [TBD]. MeSH plus Title/Abstract/Keyword terms, combined step-wise.

```
#1 MeSH descriptor: [Diabetes Mellitus, Type 2] explode all trees
#2 MeSH descriptor: [Diabetes Mellitus] explode all trees
#3 "type 2 diabetes" OR "T2DM" OR hyperglyc?emia in Title Abstract Keyword
#4 #1 OR #2 OR #3
#5 MeSH descriptor: [Periodontitis] explode all trees
#6 MeSH descriptor: [Periodontal Diseases] explode all trees
#7 periodontitis OR "periodontal disease" in Title Abstract Keyword
#8 #5 OR #6 OR #7
#9 MeSH descriptor: [Biomarkers] explode all trees
#10 biomarker* OR "MMP-8" OR "aMMP-8" OR interleukin* OR "AGE" OR "RAGE" OR microRNA* in Title Abstract Keyword
#11 #9 OR #10
#12 MeSH descriptor: [Saliva] explode all trees
#13 MeSH descriptor: [Gingival Crevicular Fluid] explode all trees
#14 saliva* OR salivary OR "gingival crevicular fluid" OR GCF in Title Abstract Keyword
#15 #12 OR #13 OR #14
#16 #4 AND #8 AND #11 AND #15
Publication Year: 1990 to 2026
```

## 6. Grey Literature

Grey-literature coverage intentionally scoped to three sources as declared in the Limitations section.

```
1. Google Scholar — first 200 results using the string:
   ("type 2 diabetes" OR "diabetes mellitus") AND (periodontitis OR "periodontal disease")
   AND (biomarker OR MMP-8 OR IL-6 OR microRNA) AND (saliva OR salivary OR "gingival crevicular fluid" OR GCF)

2. ClinicalTrials.gov — Advanced search:
   Condition: diabetes periodontitis
   Other terms: biomarker OR MMP OR interleukin OR microRNA OR saliva
   Study type: Observational | Interventional
   Completion date: 1990-01-01 to 2026-04-30

3. WHO ICTRP (trialsearch.who.int) — Advanced search:
   Condition: periodontitis AND diabetes
   Intervention or Outcome: biomarker OR MMP OR saliva
```

## Duplicate removal and record handling

All database exports (RIS/EndNote) were imported into the reference manager; duplicate records were identified and removed using exact title + author + DOI matching, followed by manual verification of fuzzy matches (same title / same first author / same year). Record counts at each PRISMA-ScR stage are reported in Figure 2 (main manuscript).
