# Supplementary material for: From biological evidence to clinical application: a scoping review of oral fluid biomarkers in type 2 diabetic periodontitis
Source: Front Oral Health. 2026 Jul 28;7:1892626. doi: 10.3389/froh.2026.1892626 (PMC13457215; doi:10.3389/froh.2026.1892626)
Supplement: Supplementary file 3 [file Table3.docx]

**Supplementary Material S3. Characteristics of Included Studies.**

| **First author, Year** | **Country** | **Design** | **Sample size (n)** | **Population / glycemic status** | **Oral fluid; assay** | **Biomarker focus (category)** |
| --- | --- | --- | --- | --- | --- | --- |
| Akram et al., 2020 | Saudi Arabia | Cross-sectional | 94 | Periodontitis with and without T2DM, and healthy controls | GCF; ELISA | AGE (glycation axis) |
| Alrabiah, 2018 | Saudi Arabia | Cross-sectional study | 90 | Prediabetes, T2DM, and non-diabetic controls with peri-implant conditions | Peri-implant sulcular fluid (PISF); ELISA | AGEs, metabolic/inflammatory (glycation-end products; endothelial/metabolic stress marker) |
| Al-Rawi et al., 2020 | UAE | Cross-sectional | 53 | DM vs non-DM ± periodontitis | Saliva; RT-qPCR | miR-146a/b, miR-155, miR-203 (microRNA panel) |
| Al-Sowygh et al., 2018 | Saudi Arabia | Cross-sectional | 93 | T2DM with peri-implant disease | Peri-implant sulcular fluid; ELISA | AGE (glycation axis) |
| Altıngöz et al., 2021 | Turkey | Cross-sectional multi-group | 121 | DM-H, DM-P, H-H, H-P | Saliva; ELISA/spectrophotometry | 8-OHdG, MDA, 4-HNE, AGE, RAGE (oxidative + glycation) |
| Aydoğdu et al., 2025 | Turkey | Cross-sectional | 64 | Healthy; Periodontitis with varying glycemic levels | Saliva, GCF; ELISA | AGE, sRAGE (glycation axis) |
| Arreguin-Cano et al., 2019 | Mexico | Cross-sectional | 97 | T2DM ± periodontitis | Saliva; gelatin zymography, Griess | MMP-2, TIMP-1, nitric oxide (tissue-destructive + oxidative) |
| Arunachalam et al., 2023 | India | Cross-sectional comparative study | 120 | Healthy, periodontitis, T2DM, and T2DM with periodontitis | Unstimulated saliva; ELISA | Inflammasomes / innate immune signaling (AIM2, IFI16); pro-inflammatory cytokine (IL-18) |
| Balaji et al., 2022 | India | Cross-sectional comparative study | 68 | Stage III/IV periodontitis with and without T2DM (HbA1c: 6.5–7.9%) | Unstimulated whole saliva; ELISA | Inflammatory markers (sTREM-1, IL-1β); tissue destruction enzyme (MMP-8) |
| Costa et al., 2010 | Brazil | Cross-sectional | 90 | controls; T2DM ± periodontitis | Whole saliva; ELISA | IL-6 (inflammatory cytokine), MMP-8 (tissue destruction/collagenase), OPG (bone metabolism regulator) |
| Diao et al., 2025 | China | Cross-sectional | 42 | T2DM with varying glycemic control | Saliva; GC-MS, 16S rRNA | Microbiome (Prevotella, Porphyromonas), Metabolome (lactate, putrescine) |
| Ding, 2021 | China | Case–control | 171 | T2DM with and without chronic periodontitis vs healthy controls | Saliva; ELISA | Developmental endothelial locus-1 (Del-1) ↓, Interleukin-17 (IL-17) ↑; TNF-α, IL-1β (inflammatory cytokines) |
| Dikilitaş et al., 2022 | Turkey | Cross-sectional comparative study | 72 | Periodontally healthy, severe periodontitis, and uncontrolled T2DM with periodontitis | GCF; ELISA | Hematopoietic growth factors / inflammatory mediators (CSF-1, IL-34) |
| Ebersole et al., 2024 | USA | Cohort | 92 | Systemically healthy non-periodontitis; T2DM ± periodontitis | Saliva; immunoassays + 16S rRNA sequencing | MMPs (MMP-8, MMP-9); Cytokines (IL-1β, IL-6, IFNα, BAFF); Adipokines (resistin); Oral microbiome |
| Ebersole et al., 2025 | USA | Cross-sectional | 92 | Adults with and without T2DM and periodontitis | Saliva; multiplex ELISA | Cytokines (IL-1β, IL-6, IFNα, BAFF); Adipokines (resistin); Oral microbiome, MMP)8/MMP9, Oral microbiome |
| Elazazy et al., 2022 | Egypt | Cross-sectional | 60 | Periodontitis with and without T2DM; healthy | GCF; RT-PCR | miR-223 (microRNA) |
| Grigoriadis et al., 2021 | Greece/Finland | Cross-sectional | 69 | Periodontal patients with normoglycemia and prediabetes | Oral rinse; aMMP-8 chairside + HbA1c POC | aMMP-8 point-of-care |
| Gupta et al., 2015 | India | Cross-sectional | 90 | T2DM ± periodontitis | Saliva; ELISA | MMP-8 (tissue-destructive enzymes) |
| Heikkinen et al., 2023 | Finland | Cross-sectional | 161 | T2DM with and without periodontitis | Oral rinse, saliva; aMMP-8 lateral flow | aMMP-8 point-of-care |
| Kajiura et al., 2014 | Japan | Cross-sectional | 78 | T2DM with and without periodontitis; Periodontitis; Healthy | GCF; ELISA | Glycated albumin, calprotectin |
| Koregol et al., 2018 | India | Case–control | 90 | Periodontitis with and without T2DM, healthy controls | Saliva; 8-IsoP immunoassay | Oxidative stress (lipid peroxidation marker: 8-isoprostane) |
| Liu et al., 2021 | China | Cross-sectional | 97 | Periodontitis with and without T2DM; healthy with and without T2DM | GCF; RT-PCR | miR-223, miR-200b (microRNA panel) |
| Miller et al., 2021 | USA | Case-control | 61 | T2DM ± periodontitis | Saliva; ELISA / multiplex | MMP-8, IL-1β, IL-6, resistin (composite panel) |
| Miller et al., 2026 | USA | Secondary analysis | 92 | T2DM ± periodontitis | Saliva; immunoassay + 16S rRNA | Host-microbe multi-marker panel |
| Mohamed et al., 2015 | Sudan | Cross-sectional comparative study | 152 | T2DM with and without chronic periodontitis vs non-diabetic controls | GCF; multiplex biometric immunoassay | Glucoregulatory biomarkers (GIP, GLP-1, glucagon, C-peptide, insulin, ghrelin, leptin, PAI-1, visfatin) – metabolic/periodontal interaction markers |
| Oruc & Babayigit, 2026 | Turkey | Cross-sectional | 88 | Diabetic and non-diabetic ± periodontitis | Unstimulated saliva; ELISA | IL-1β, IL-39, IL-40, asprosin (cytokines/adipokines) |
| Peniche-Palma et al., 2019 | Mexico | Cross-sectional | 80 | T2DM± periodontitis | GCF; Multiplex/Luminex | MMP-9 (host enzyme), MPO (oxidative stress) |
| Pradeep et al., 2013 | India | Cross-sectional | 40 | Periodontitis with and without T2DM and healthy controls | GCF; spectrophotometry | 4-HNE, MDA (oxidative stress) |
| Sangappa et al., 2024 | India | Cross-sectional | 120 | T2DM with chronic periodontitis ± tooth loss | Unstimulated saliva; ELISA | IL-6 (inflammatory cytokine) |
| Saxena et al., 2020 | India | Cross-sectional | 85 | T2DM with periodontitis and healthy controls | GCF; ELISA | IL-17, Del-1 (IL-17/Del-1 axis) |
| Tavakoli et al., 2024 | USA | Cross-sectional | 98 | T2DM ± periodontitis; HbA1c reported | Saliva; ELISA | MMP-8 (tissue-destructive enzymes) |
| Techatanawat et al., 2020 | Thailand | Cross-sectional | 49 | T2DM with periodontitis | Saliva; ELISA | IL-17A, IL-18 (cytokines) |
| Thomas et al., 2025 | India | Cross-sectional diagnostic accuracy | 98 | T2DM ± periodontitis with varying glycemic control | saliva; ELISA | aMMP-8, AGE, RAGE (tissue-destructive + glycation) |
| Thomas JT, 2025 | India | Cross-sectional | 77 | MetS and/or periodontitis | Oral rinse, saliva, GCF; ELISA | aMMP-8 (tissue destruction / matrix metalloproteinase biomarker) |
| Tk F et al., 2022 | India | Case–control | 72 | Periodontitis with and without T2DM, healthy controls | GCF; ELISA (sulfiredoxin), colorimetric assay (TOS) | Oxidative stress & antioxidant response (TOS, sulfiredoxin) |
| Trivedi et al., 2014 | India | Cross-sectional | 60 | Periodontitis with and without T2DM; healthy with and without T2DM | Saliva; ELISA/enzymatic | MDA, SOD, CAT (oxidative + antioxidant) |
| Umeizudike et al., 2025 | Greece | Cross-sectional | 150 | Periodontitis patients and prediabetes | Oral rinse, saliva; IFMA | aMMP-8, MMP-9, TIMP-1 (tissue-destructive enzymes) |
| Vincent et al., 2018 | India | Cross-sectional | 80 | T2DM ± periodontitis | Saliva; enzymatic assays | SOD, CAT, GR (antioxidant enzymes) |
| Yilmaz et al., 2020 | Turkey | Cross-sectional case–control study | 178 | T2DM and non-diabetic individuals; with and without periodontitis; includes smokers | Unstimulated whole saliva; ELISA | Antimicrobial peptides / innate immunity (hBD-1, hBD-2, hBD-3, LL-37); metabolic marker (AGEs) |
| Yoon et al., 2012 | USA | Cross-sectional | 192 | T2DM and/or periodontitis | Unstimulated saliva; β-glucuronidase (fluorometric assay), IL-1β (ELISA) | Inflammatory burden markers (innate immune enzymes and cytokines) |

**Abbreviations**

AGEs: Advanced glycation end products; AIM2: Absent in melanoma 2; aMMP-8: active matrix metalloproteinase-8; BAFF: B-cell activating factor; BOP: bleeding on probing; CAL: clinical attachment level; CAT: catalase; CP: chronic periodontitis; CSF-1: colony stimulating factor-1; Del-1: developmental endothelial locus-1; DM: diabetes mellitus; ELISA: enzyme-linked immunosorbent assay; FBG: fasting blood glucose; GCF: gingival crevicular fluid; GC-MS: gas chromatography–mass spectrometry; GIP: glucose-dependent insulinotropic polypeptide; GLP-1: glucagon-like peptide-1; GR: glutathione reductase; HbA1c: glycated hemoglobin; hBD: human beta-defensin; IFMA: immunofluorometric assay; IFNα: interferon alpha; IFI16: interferon gamma inducible protein 16; IL: interleukin; LL-37: cathelicidin antimicrobial peptide; MDA: malondialdehyde; miR/miRNA: microRNA; MIP-1α/β: macrophage inflammatory protein-1 alpha/beta; MMP: matrix metalloproteinase; MPO: myeloperoxidase; MetS: metabolic syndrome; NF-κB: nuclear factor kappa B; NO: nitric oxide; OPG: osteoprotegerin; PAI-1: plasminogen activator inhibitor-1; PD: periodontal disease; PISF: peri-implant sulcular fluid; PMN: polymorphonuclear neutrophils; RAGE: receptor for advanced glycation end products; RANKL: receptor activator of nuclear factor kappa-B ligand; RANTES: regulated upon activation, normal T-cell expressed and secreted; ROS: reactive oxygen species; RT-PCR: reverse transcription polymerase chain reaction; sRAGE: soluble receptor for advanced glycation end products; SOD: superoxide dismutase; T2DM: type 2 diabetes mellitus; TAOC: total antioxidant capacity; TIMP-1: tissue inhibitor of metalloproteinases-1; TOS: total oxidative stress; TNF-α: tumor necrosis factor alpha; VEGF: vascular endothelial growth factor; WSS: whole saliva sample; βG: beta-glucuronidase.
